# Highly competent, non-exhausted CD8+ T cells continue to tightly control pathogen load throughout chronic *Trypanosoma cruzi* infection

**DOI:** 10.1371/journal.ppat.1007410

**Published:** 2018-11-12

**Authors:** Angela D. Pack, Matthew H. Collins, Charles S. Rosenberg, Rick L. Tarleton

**Affiliations:** 1 Department of Microbiology, University of Georgia, Athens, Georgia, United States of America; 2 Center for Tropical and Emerging Global Diseases, University of Georgia, Athens, Georgia, United States of America; 3 Department of Cellular Biology, University of Georgia, Athens, Georgia, United States of America; Queensland Institute of Medical Research, AUSTRALIA

## Abstract

*Trypanosoma cruzi* infection is characterized by chronic parasitism of non-lymphoid tissues and is rarely eliminated despite potent adaptive immune responses. This failure to cure has frequently been attributed to a loss or impairment of anti-*T*. *cruzi* T cell responses over time, analogous to the T cell dysfunction described for other persistent infections. In this study, we have evaluated the role of CD8^+^ T cells during chronic *T*. *cruzi* infection (>100 dpi), with a focus on sites of pathogen persistence. Consistent with repetitive antigen exposure during chronic infection, parasite-specific CD8^+^ T cells from multiple organs expressed high levels of KLRG1, but exhibit a preferential accumulation of CD69^+^ cells in skeletal muscle, indicating recent antigen encounter in a niche for *T*. *cruzi* persistence. A significant proportion of CD8^+^ T cells in the muscle also produced IFNγ, TNFα and granzyme B *in situ*, an indication of their detection of and functional response to *T*. *cruzi in vivo*. CD8^+^ T cell function was crucial for the control of parasite burden during chronic infection as exacerbation of parasite load was observed upon depletion of this population. Attempts to improve T cell function by blocking PD-1 or IL-10, potential negative regulators of T cells, failed to increase IFNγ and TNFα production or to enhance *T*. *cruzi* clearance. These results highlight the capacity of the CD8^+^ T cell population to retain essential *in vivo* function despite chronic antigen stimulation and support a model in which CD8^+^ T cell dysfunction plays a negligible role in the ability of *Trypanosoma cruzi* to persist in mice.

## Author summary

The parasite *Trypanosoma cruzi* establishes lifelong infections in humans and other mammals, leading to severe cardiac and gastrointestinal complications known as Chagas disease. Although the factors that enable *T*. *cruzi* persistence remain undefined, in this and many other infection models, pathogen persistence has been attributed to the exhaustion of the immune system, particularly of CD8^+^ T cells. Here, we show that the inability of hosts to fully resolve *T*. *cruzi* infection is not a result of immune exhaustion and that in fact the *T*. *cruzi*-specific CD8^+^ T cell population remains highly competent and actively suppresses parasite outgrowth throughout the chronic infection. These results demonstrate that compromised immunity is not the eventual outcome of all chronic infections and suggest that *T*. *cruzi*, and perhaps other pathogens, may employ alternative strategies to subvert immune clearance in the presence of highly functional pathogen-specific effectors. These findings also suggest that interventions to inhibit immune regulatory pathways or to otherwise boost existing immune responses in such infections, will have limited benefit.

## Introduction

Chagas disease, caused by the protozoal parasite *Trypanosoma cruzi*, is the leading cause of infectious myocarditis globally and affects at least 10 million individuals worldwide [1, 2]. Over decades of infection, many infected individuals develop clinical disease including megacolon and dilated cardiomyopathy for which treatment options are limited [3]. The pathophysiology of Chagas disease is best described as chronic inflammatory damage driven by persistent parasitism of affected tissues. A better understanding of the immune mechanisms regulating the host-parasite interaction and disease outcomes is needed if improved therapeutic and preventive strategies for this neglected tropical disease are to be developed.

Immune control of intracellular pathogens like *T*. *cruzi* is dependent on MHC class I presentation of cytoplasmic antigens (Ag) and the subsequent destruction of infected cells as a result of inflammatory cytokine production or cytolysis by CD8^+^ T cells [4, 5]. In many infections, effective immunity results in acute phase pathogen clearance, with recognition and elimination of infected host cells early in the infection cycle, thus preventing pathogen spread and contributing to rapid infection resolution. During infections where complete pathogen clearance does not occur, or is significantly delayed, persistent antigen can drive the emergence of ‘exhausted’ T cells with diminished capacity to produce key cytokines and reduced replicative potential, and in extreme cases, T cell deletion by apoptosis [6–8]. In some instances, this exhausted state is reversible by interrupting one or more of a number of regulatory mechanisms responsible for restraining CD8^+^ T cell activity, e.g. regulatory T cells (Tregs), inhibitory cytokines, or inhibitory receptors such as programmed cell death-1 (PD-1) [9]. While these regulatory programs minimize immunopathology, they may also compromise infection resolution [10–13].

CD8^+^ T cells are essential for host survival of acute *T*. *cruzi* infection [14, 15], but the significance of this population in control of chronic infection is not well defined. The high parasite load characteristic of acute *T*. *cruzi* infection, is ultimately reduced to nearly undetectable levels in most hosts, a feat that involves, and in fact depends upon the extensive expansion and effector function of anti-*T*. *cruzi* CD8^+^ T cells [16]. Based upon the evidence for persistence of *T*. *cruzi* in infected hosts and the attribution of parasite persistence to impaired/exhausted T cells [17, 18] as well as the demonstration that persistent Ag stimulation is associated with reduced T cell function in other systems including several intracellular protozoans [19–21], investigation of the contribution of T cell exhaustion in *T*. *cruzi* persistence is warranted.

The lack of direct evidence regarding the *in vivo* contribution of CD8^+^ T cells to control of chronic *T*. *cruzi* infection and the emergence of reports questioning the validity of *ex vivo* assays to predict *in vivo* T cell function [22–24], prompted us to attempt to conclusively determine whether compromised CD8^+^ T cell function is the primary factor enabling the persistence of *T*. *cruzi*. For this, we examined the stability and *in vivo* functional capacity of *T*. *cruzi*-specific cells in spleen and skeletal muscle during chronic *T*. *cruzi* infection and evaluated the relative contribution of immunoregulation in the forms of PD-1 and IL-10 to T cell activity and parasite persistence. Our findings indicate that despite chronic antigen stimulation, CD8^+^ T cells continue to exert critical effector functions to limit parasite expansion throughout the course of *T*. *cruzi* infection.

## Results

### Pathogen-specific tissue-resident T cells exhibit markers of recent antigen exposure

**Fig 1:**
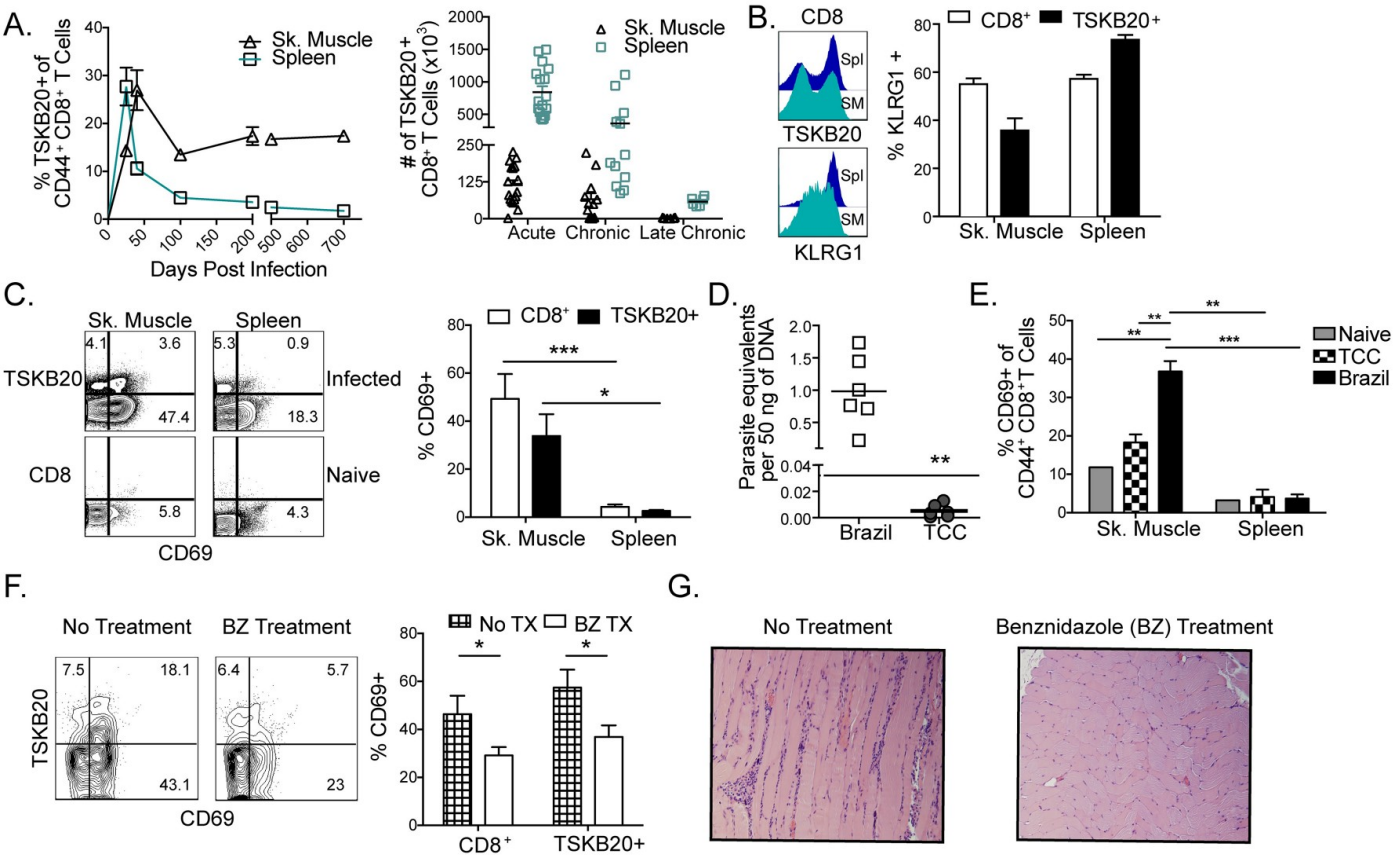
Parasite-specific CD8^+^ T cells encounter antigen and express markers of activation during chronic infection. (A) The frequency (left) and number (right) of CD8^+^ T cells specific for the immunodominant epitope, TSKB20, in the pool of all CD44^+^ CD8^+^ T cells was measured longitudinally in spleen (open square) and skeletal muscle (open triangle). (B) Representative histograms of the KLRG1 expression in the total CD8^+^ (top) and TSKB20+ (bottom) populations. CD44^hi^ KLRG1+ cells detected in the total (white bar) and parasite-specific (black bar) CD8^+^ T populations in the spleen and muscle at 150 dpi. (C) Recently activated CD8^+^ T cells are enriched in skeletal muscle. Representative flow plots show surface expression of CD69 for cells isolated from indicated tissue during chronic (150 dpi) *T*. *cruzi* infection. Numbers describe the percentage of cells expressing CD69 in each gate from the total CD44+ population. Summary data of the percentages of CD69+ cells in total (white) and TSKB20+ (black) bars are displayed graphically to the right. (D) Quantification of chronic parasite burden in skeletal muscle via qPCR during chronic (>250 dpi) Brazil and TCC strain *T*. *cruzi* infection. The line represents the limit of detection for qPCR. Persistence of the TCC strain has been previously confirmed via qPCR and hemoculture following immunosuppression [86] (E) The frequency of CD8^+^ T cells that express CD69 in the muscle of mice chronically infected is highest in those infected with Brazil strain relative to the low level but persistent, TCC strain. (F) Reduction in parasite burden following short-term benznidazole treatment results in decreased CD69 expression and contraction of parasite-specific T cells in the muscle during chronic infection (>250 dpi). (G) Antigen-dependent inflammation observed during chronic infection is reduced following sub-curative (19d) treatment with the trypanocidal compound benznidazole (BZ). Skeletal muscle was fixed in 10% formalin, embedded in paraffin, sectioned, and stained with H&E. All data are representative of at least three independent experiments with n = 3–5 with the exception of panels D-G which are representative of two independent experiments. Data are mean + SEM. * indicates percentage levels that are significantly different (* P ≤ 0.05, ** P ≤ 0.01,***P ≤ .001) between specified groups.

Parasite levels in blood and in tissues during *T*. *cruzi* infection tend to peak between 15 and 30 days of infection and thereafter, become essentially undetectable in blood and increasingly restricted in distribution in tissues, where they are demonstrable only with highly sensitive amplification techniques [25]. The CD8^+^ T cell response to *T*. *cruzi* during the acute and chronic stages is nearly monopolized by T cells recognizing strongly immunodominant epitopes from trans-sialidase family proteins [26]. In B6 mice, TSKB20 (ANYKFTLV)–specific T cells expand to very high levels during acute *T*. *cruzi* infection before contracting and remaining at relatively lower levels as the infection is controlled (Fig 1A). In the skeletal muscle, a site of *T*. *cruzi* persistence, TSKB20-specific T cells maintain a stable, high fraction of the total Ag-experienced CD8^+^ population throughout acute and chronic infection, although total numbers of such cells decline in concert with declining parasite load. Both splenic and skeletal muscle *T*. *cruzi*-specific CD8^+^ T cells exhibited substantial KLRG1 expression well into the chronic infection, indicative of prior Ag exposure (Fig 1B). Furthermore, only in tissues of parasite persistence, including skeletal muscle (Fig 1C), heart, and fat (S1 Fig), was the marker of recent activation, CD69, expressed on a substantial proportion of *T*. *cruzi*–specific CD8^+^ T cells.

CD69 can also serve as a marker for tissue residence of T cell populations [27] so we further explored the dependence of its expression on antigen load. The activation status and accumulation of *T*. *cruzi-*specific T cells was related to parasite antigen abundance in the chronically infected tissues, as shown by the fact that CD69 expression was decreased in the case of infection with the low level persistent TCC strain (Fig 1D and 1E) and in mice treated with a non-curative course of the trypanocidal compound benznidazole (BZ) (Fig 1F). Recent studies have shown that a single dose of BZ reduces parasite load by ~90% and 6 daily doses, by 3 log_10_ [28]. Here, as also previously reported [29], reduction of parasite numbers via a 19 day course of BZ treatment resulted in overall fewer inflammatory infiltrates (Fig 1G). Collectively, these data provide strong evidence that *T*. *cruzi*-specific T cells regularly encounter and are responsive to parasites at sites of pathogen persistence in chronically infected hosts.

### *T*. *cruzi*-specific CD8^+^ T cells exhibit potent effector functions *in vivo* during chronic infection

**Fig 2:**
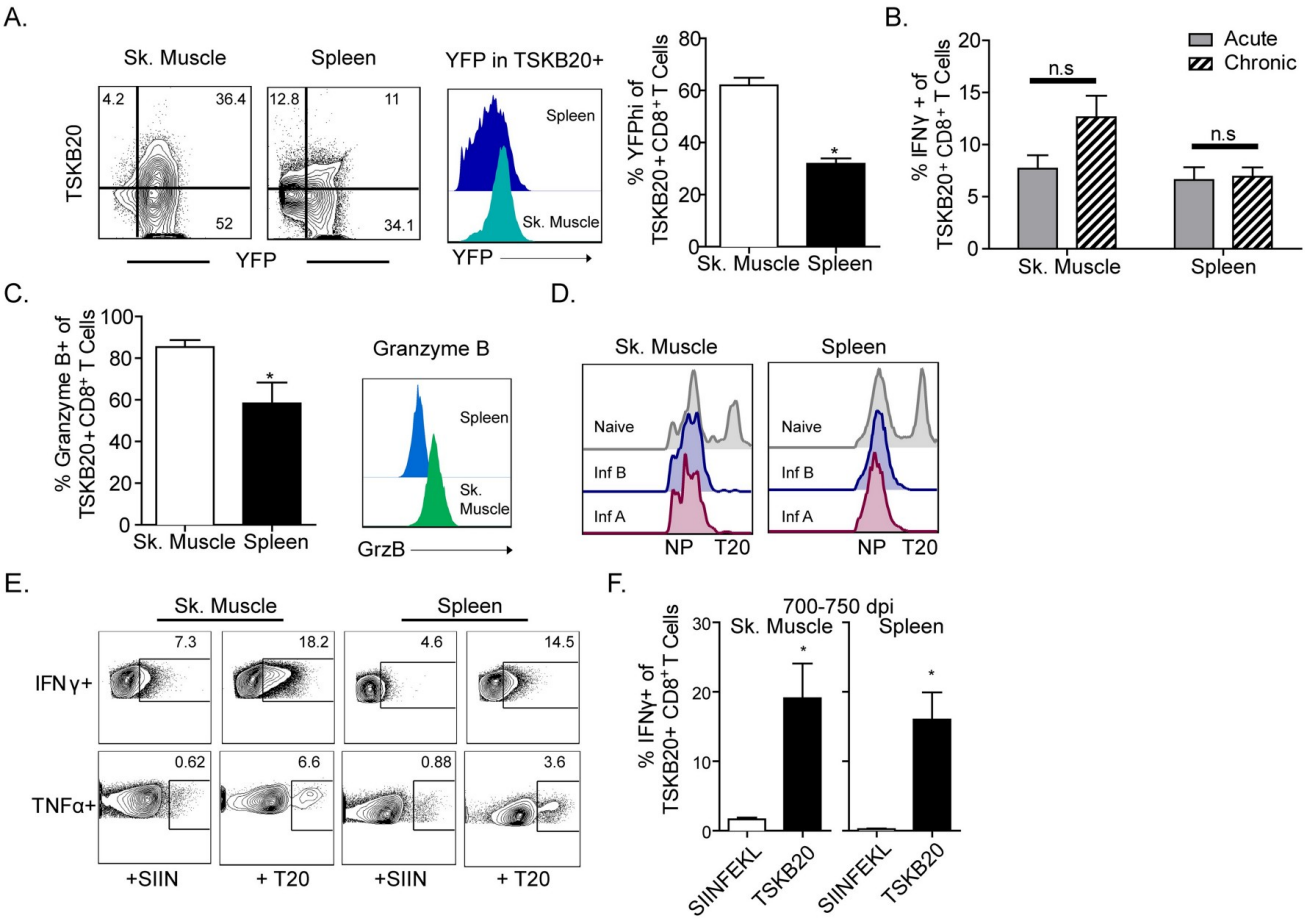
*T*. *cruzi*-specific CD8^+^ T cells continue to produce effector molecules during chronic infection. (A) IFNγ production by *T*. *cruzi*–specific T cells in the spleen and muscle at 90 dpi and in naïve mice was assessed using an eYFP IFNγ transcription reporter model. Representative flow plots (left) of the frequency of YFP positive cells in the CD44+ population in the muscle and spleen and histograms describe YFP expression by TSKB20+ CD8^+^ cells. Column graphs describe the frequency of YFP^hi^ cells in the TSKB20+ population. (B) Mice were injected with brefeldin A and approximately 6 hours later, tissues were harvested and stained to assess *in situ* cytokine production. Bar graphs describe IFNγ production by TSKB20+ CD8^+^ T cells during acute (<40d) or chronic (200d) infection directly *ex vivo* without additional stimulation. (C) Granzyme B production was measured directly *ex vivo* in parasite-specific CD8^+^ T cells during chronic infection (200–250 dpi) and is displayed and summarized in column graphs (left) and representative histograms (right). (D) CD8^+^ T cells from the muscle and spleen continue to perform CTL activity *in vivo* at >250 dpi. Mice were injected with CFSE labeled TSKB20-peptide pulsed and un-pulsed splenocytes and target cell lysis was assessed ~18 hours later. (E) Mice were injected simultaneously with brefeldin A and either TSKB20 or SIINFEKL (control) peptide epitopes prior to tissue collection 6–8 hours later. Cytokine production (IFNγ and TNFα) was evaluated without *ex vivo* stimulation at ~200 dpi. (F) *T*. *cruzi*-specific cells remain responsive to specific *in vivo* peptide stimulation even during late chronic infection (700–750 dpi). The frequency of IFNγ producing TSKB20+ CD8^+^ T cells increases significantly upon *in vivo* TSKB20 peptide administration in the muscle and spleen. Data are mean + SEM and are representative of 3–5 independent experiments with n = 3, with the exception of D and F, which are representative of two experiments. * indicates values that are significantly different (* P ≤ 0.05, ** P ≤ 0.01) between experimental and control peptide recipients.

We next assessed the quality of the effector functions of *T*. *cruzi*-specific T cells responding to parasites in peripheral tissues of mice with chronic *T*. *cruzi* infection. IFNγ production, measured directly *ex vivo* without restimulation with both a YFP-reporter mouse strain (Fig 2A) and intracellular cytokine staining (Fig 2B), demonstrated that *T*. *cruzi*-specific CD8^+^ T cells were significantly more activated for production of this critical cytokine in peripheral sites of parasite persistence (skeletal muscle) as compared to the spleen. Additionally, a higher frequency of CD8^+^ T cells in the skeletal muscle showed granzyme B expression (Fig 2C), a measure of CD8^+^ T cell capacity to eliminate infected target cells through the delivery of cytotoxic granules, relative to splenic counterparts. Consistent with the observation that *T*. *cruzi*-specific CD8^+^ T cells retain their cytolytic function in the chronic phase of infection, in vivo CTL assays showed that trans-sialidase peptide-sensitized splenocytes were depleted with high efficiency in infected animals (Fig 2D).

To evaluate further the capacity of parasite-specific T cells in peripheral tissues to respond to parasite Ag *in vivo* during chronic *T*. *cruzi* infection, mice were injected with control (SIINFEKL) or *T*. *cruzi* (TSKB20) peptide epitopes and assessed for cytokine production by ICS. CD8^+^ T cells responded to specific peptide stimulation with significant increases in cytokine production (IFNγ and TNFα) in both the spleen and skeletal muscle (Fig 2E). This effector function extended beyond the “early” (150–250 dpi) chronic phase, as CD8^+^ T cells in both the muscle and spleen retained the capacity to respond to parasite epitope injection with the production of IFNγ at >700 days post infection (Fig 2F). Thus, coincident with reduced antigen levels at these late chronic time points, basal *in situ* cytokine production was low compared to earlier in the infection, but a significant proportion of the *T*. *cruzi*-specific T cell population retained the capacity to produce this key inflammatory cytokine when re-encountering antigen.

### CD8^+^ T cell function is required for maintenance of parasite control in chronic *T*. *cruzi* infection

**Fig 3:**
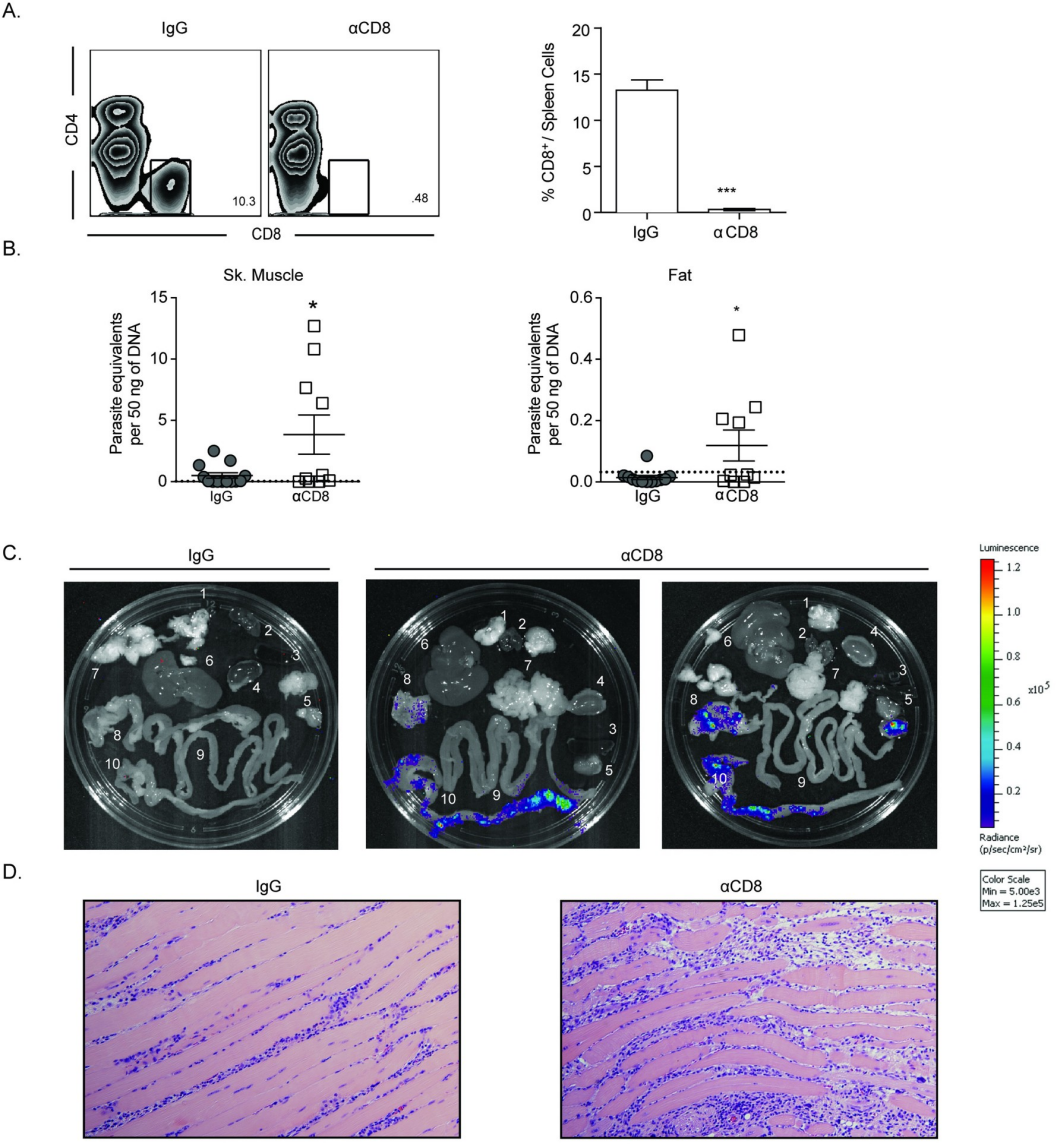
CD8^+^ T cell function is required for optimal control of chronic *T*. *cruzi* infection. Chronically infected mice (>100 dpi) were treated for 21d with αCD8 or IgG control antibody. (A) A representative flow plot of the percentage of CD8^+^ T cells remaining in the splenic lymphocyte population following CD8 depletion (left) and graphical representation of depletion efficacy in the groups (right). (B) Depletion of CD8^+^ T cells during chronic infection results in increased parasite burden in tissues of persistence: muscle (left) and fat (right). The dashed line represents the limit of detection for quantitative real-time PCR. (C) CD8 depletion results in parasite outgrowth in tissues of persistence, stomach (8), large intestine (10), and skeletal muscle (5) in mice chronically infected with luciferase-expressing Colombiana strain parasites. The tissues included in the *ex vivo* luciferase assay are as follows: brain (1), heart (2), spleen (3), kidney (4), skeletal muscle (5), liver (6), mesenteric fat (7), stomach (8), small intestine (9), and large intestine (10). (D) H&E staining of skeletal muscle reveals that CD8 depletion exacerbates tissue inflammation and results in deterioration of tissue integrity. All data are representative of two independent experiments with n = 5 with the exception of panel C. Data are mean + SEM. * indicates values that are significantly different (* P ≤ 0.05, ** P ≤ 0.01, ***P ≤ 0.001) between compared groups.

The data presented to this point indicate that *T*. *cruzi*–specific T cells at sites of parasite persistence are capable of sensing and responding to *T*. *cruzi*. To evaluate whether these CD8^+^ T cells provide a host-protective function in the chronic phase of infection, anti-CD8 antibody was administered to mice beginning at >100 dpi. Treatment significantly reduced circulating CD8^+^ T cells (Fig 3A) when compared to IgG-treated controls and resulted in significantly higher parasite load in skeletal muscle and fat in the CD8-depleted group (Fig 3B). Monitoring of luciferase-expressing parasites in intact organs also demonstrated the substantial increase in parasite levels, particularly in the gastrointestinal tract, in mice depleted of CD8^+^ T cells (Fig 3C). Notably, depletion of CD8^+^ T cells also resulted in increased severity of inflammation and disruption of tissue integrity in skeletal muscle (Fig 3D). Thus, tissue-homing *T*. *cruzi*–specific CD8^+^ T cells retain important effector functions and are vital to infection control in chronic *T*. *cruzi* infection.

### Maintenance of CD8^+^ T cell response to *T*. *cruzi* in peripheral tissue

**Fig 4:**
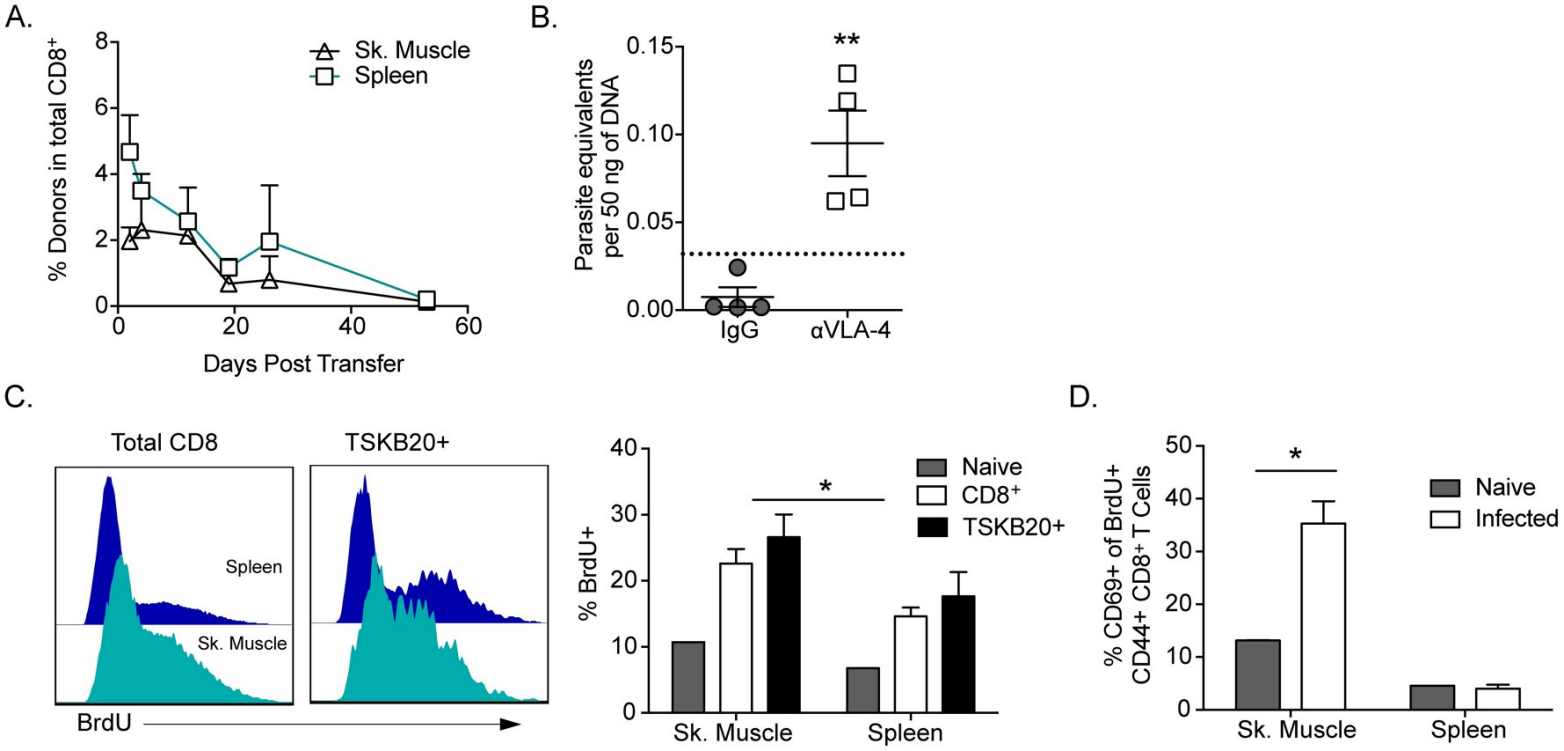
Trafficking and maintenance of CD8^+^ T cell response to *T*. *cruzi* in peripheral tissue. (A) CD8^+^ T cells are recruited from a central pool to non-lymphoid tissues in chronic *T*. *cruzi* infection. CD8^+^ T cells were magnetically purified from infection-matched CD45.1+ donor mice (>100 dpi) and transferred into CD45.2+ recipient mice. Cells were isolated from spleen (square) and skeletal muscle (triangle) at various time points after transfer. Mean ± SEM of two samples analyzed at each time point is shown and represents data obtained in three independent experiments. (B) Interruption of leukocyte trafficking is detrimental to parasite control. Mice (>100 dpi) were treated with a blocking anti-VLA-4 Ab for 30 days, and parasite load was determined in skeletal muscle. * indicates p<0.05 compared to control by Mann-Whitney test. A similar trend (p<0.05) was observed in 2 of 3 additional experiments. (C) Recently replicated CD8^+^ T cells are enriched in tissues of parasite persistence. Mice chronically infected with *T*. *cruzi* were given BrdU in their drinking water for 21 days. Lymphocytes were then isolated from lymphoid and non-lymphoid tissue. Histograms describe BrdU incorporation by splenic (blue) and muscle-derived (teal) CD8^+^ T cells in the total (left) and TSKB20+ (right) population. These data are summarized in accompanying column graph (right). (D) Dividing CD8^+^ T cells develop into activated effectors at sites of parasite persistence. Expression of CD69 was examined among proliferating (BrdU+) CD8^+^ T cells in muscle and spleen in naïve animals and mice with chronic *T*. *cruzi* infection on day 21 of BrdU administration. Data in panels C and D are mean + SEM and are representative of two independent experiments with n = 4–5. * indicates values that are significantly different (* P ≤ 0.05, ** P ≤ 0.01, ***P ≤ 0.001) between compared groups.

We next explored the question of whether T cells in the peripheral tissue sites of parasite persistence are being continuously recruited from the systemic population. CD8^+^ T cells from the spleens of chronically *T*. *cruzi*-infected donor mice (CD45.1) were transferred to infection-matched congenic recipient (CD45.2) mice. Donor cells were promptly incorporated into the CD8^+^ T cell population at sites of parasite persistence as early as 2 days post-transfer and are detectable in the tissue for up to 50 days after transfer (Fig 4A). These data show that even in the setting of low-level chronic inflammation, CD8^+^ T cell trafficking is a dynamic process, and CD8^+^ T cells responding to *T*. *cruzi* move readily from the circulation into tissues harboring parasite-infected cells. To determine if frequent recruitment of effector cells to infected tissue was a key mechanism for controlling *T*. *cruzi* during chronic infection, trafficking of T cells was disrupted by administering a blocking antibody to VLA-4, a molecule involved in leukocyte extravasation [30]. Mice receiving anti-VLA-4 antibody exhibited an increased parasite load in skeletal muscle, indicating that infected tissues depend on continuous leukocyte trafficking to control parasite growth (Fig 4B).

In addition to continued recruitment of Ag-experienced CD8^+^ T cells into responses to persistent pathogens, recruitment of recently activated T cells during an ongoing immune response may be necessary to sustain effector cell levels. To determine whether new parasite-specific effectors are generated during chronic *T*. *cruzi* infection, the level of recently replicated (BrdU+) cells was assessed following *ad libitum* BrdU administration for 21 days. A substantial fraction of CD8^+^ and TSKB20^+^ cells in muscle and spleen were BrdU+, with an increased accumulation in skeletal muscle (Fig 4C). Many of these recently replicated cells become exposed to *T*. *cruzi* antigen and possessed an effector activation phenotype, with substantial numbers expressing CD69 in the muscle of infected animals (Fig 4D). These data support a model of a very active immune response requiring T cell migration into parasite-infected tissues, and one in which T cell replication continues to occur despite chronic antigen stimulation.

### Multiple immune checkpoints have a negligible impact on *T*. *cruzi* persistence

**Fig 5:**
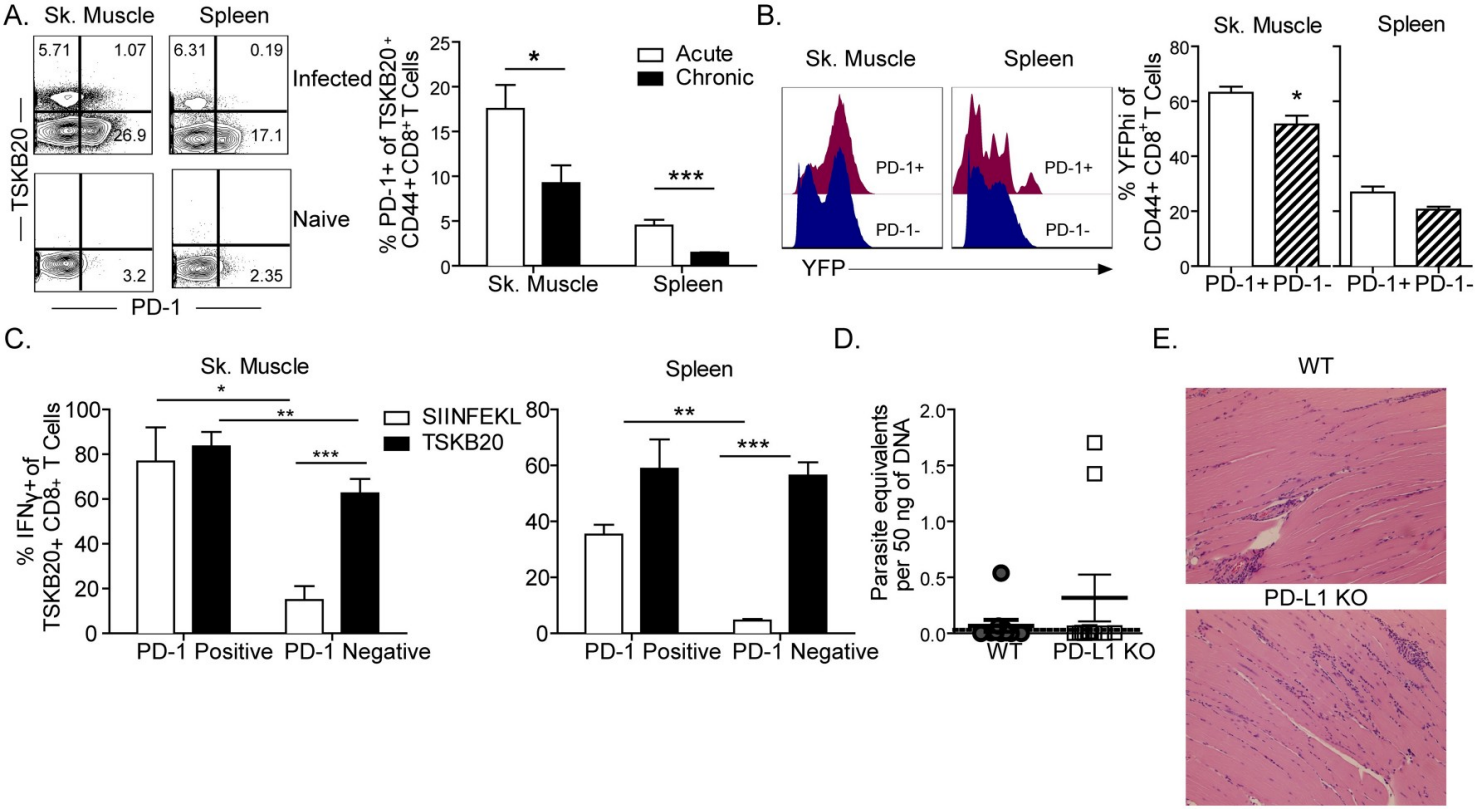
CD8^+^ T cells are not regulated by a PD-1-dependent mechanism in chronic *T*. *cruzi* infection. (A) The proportion of PD-1+ parasite-specific CD8^+^ spleen and muscle derived cells decreases between acute (<40d) and chronic infection (>100d). Representative flow plots of PD-1 expression in CD44^+^ CD8+ T cells in the tissues of infected and naïve mice are provided (left) and changes in PD-1 expression across time in the parasite-specific population are summarized graphically. (B) Comparable portions of PD-1 positive and negative cells produce IFNγ at 90 days post-infection, as indicated by YFP expression, in muscle (left) and spleen (right). The frequency of YFP^hi^ cells in the total CD8^+^ population in the muscle and spleen are shown. (C) PD-1 expression does not undermine the capacity of CD8^+^ T cells to respond to antigen stimulation *in vivo* in skeletal muscle (left) or spleen (right) during chronic infection. (D) Mice lacking PD-L1 do not exhibit enhanced clearance of *T*. *cruzi* infection. Parasite load in skeletal muscle of PD-L1 KO and WT mice during chronic (167 dpi) *T*. *cruzi* infection was assessed by qPCR. The dashed line represents the limit of detection. (E) PD-L1 KO mice experience levels of inflammation similar to WT mice during chronic *T*. *cruzi* infection. H&E sections of skeletal muscle from infected WT and PD-L1 KO mice. Graphs represent mean + SEM. * indicates values that are significantly different (* P ≤ 0.05, ** P ≤ 0.01) between compared groups. All data are representative of at least two independent experiments with minimum n = 3.

The retention of effector function and participation in infection control of CD8^+^ T cells in mice with chronic *T*. *cruzi* infection is in contrast to the often-reported loss of the quality of effector responses in multiple other chronic infectious diseases [31–33]. In a number of these other models, checkpoint molecules such as PD-1 have been implicated to have a negative impact on pathogen clearance [21, 34, 35]. Further, blockade of PD-L1 (and to a much lesser extent PD-L2), and PD-1 knockout (KO) has been shown to increase inflammatory responses and mortality in acute *T*. *cruzi* infection [18]. To explore the potential role of PD-1 in constraining T cell responses and supporting parasite persistence during chronic *T*. *cruzi* infection, we carefully examined PD-1 expression on *T*. *cruzi*-specific CD8^+^ T cells during chronic infection. Numbers of PD-1 expressers was always significantly higher in the muscle relative to the spleen, consistent with PD-1 being a marker of antigen activation (Fig 5A) as reported in other systems [36, 37]. Assessing direct *ex vivo* IFNγ responses confirmed that PD-1 expressing cells were not compromised for infection- or epitope-induced cytokine production (Fig 5B and 5C). Indeed, the frequency of PD-1 expressers spontaneously producing IFNγ was already quite high (~70%), which left little room for increased production upon exposure to additional specific epitope stimulation. LAG3 or TIM3, additional markers of T cell exhaustion, were also expressed at very low frequency in total or *T*. *cruzi*–specific CD8^+^ T cells in late chronic infection (>500 dpi; S2A and S2B Fig).

To address directly the impact of PD-L1 on control of parasite burden, parasite load and tissue inflammation were evaluated in PD-L1 KO mice. Preventing PD-1:PD-L1 engagement failed to improve parasite control (Fig 5D) or alter tissue pathology (Fig 5E) during chronic infection, although the already low parasite burden in these tissues in chronically infected animals made detection of improved parasite control nearly impossible. Extended *in vivo* blockade of PD-L1 by antibody treatment also failed to enhance the capacity of CD8^+^ T cells in the muscle or spleen to produce inflammatory cytokines in response to polyclonal *ex vivo* stimulation (S3A Fig). Experiments to counter the potential activity of interleukin-10 (IL-10), another major regulator of T cell responses in chronic infections [12, 38, 39], yielded no evidence of a change in parasite load during either the acute (in IL-10KO mice; S4A and S4B Fig) or in chronically infected mice (using anti-IL-10 receptor (IL-10R) antibody; S4C Fig) although again the characteristically low parasite load in chronically infected mice made the impact of blocking of IL-10 function at this time point, inconclusive. Taken together, these data provide no evidence for the generation of T cell exhaustion or other regulatory mechanisms in limiting the function of CD8^+^ T cell responses in chronic *T*. *cruzi* infection in mice.

## Discussion

Persistent infections present special challenges for the immune system and the cause of pathogen persistence is often not clear; is it the result of failed or regulated anti-pathogen immune responses or is it an inherent property of a host-pathogen interaction, maintained, for example, by pathogen immune evasion mechanisms? In the latter case, persistence may or may not ultimately result in antigen-driven regulation of T cell responses.

The primary goal of this study was to determine the functional status of CD8^+^ T cells during chronic *T*. *cruzi* infection and to assess the contribution of these cells to long-term control of persistent parasites. *T*. *cruzi* infection is thought to persist for the life of hosts, though there are a few anecdotal reports of apparent spontaneous cure in humans [40–42] and in mice (a natural host for this parasite) [43]. The lack of parasite clearance in *T*. *cruzi* infection has been attributed to sub-par or highly regulated immune responses [17, 18, 44, 45] although a direct role for such regulation in preventing parasite clearance is lacking. We were particularly interested in evaluating anti-parasite CD8^+^ T cell responses at the host-pathogen interface (in parasite-infected tissues), as this cell population in these sites is most likely to be exposed continuously to parasite antigen and tissue inflammation and thus most likely vulnerable to becoming exhausted. We hypothesized that *T*. *cruzi*-specific CD8^+^ T cells retain critical effector function, and that suppression of *T*. *cruzi* to very low levels is actually a “successful” immunologic outcome that simultaneously limits sequelae of an uncontrolled infection and immunopathology in vital host tissues. This hypothesis is contrary to the paradigm that impaired pathogen clearance is primarily associated with alterations in and regulation of host immunity [19, 46–48].

Here, we have demonstrated that the effector CD8^+^ T cell response is very robust, highly functional and is essential for parasite control during chronic *T*. *cruzi* infection, despite persistent antigen activation. Persistent antigen activation is demonstrated by the expression of surface markers indicative of recent antigen exposure on, as well as by the direct *ex vivo* detection of cytokine production by T cells from tissues known to harbor persistent parasites. Functionality is demonstrated by these same immunological traits, by the ability of a subset of the T cells to respond *in vivo* to a *T*. *cruzi* peptide epitope, and by the essentiality of these cells in containing parasite load in these same tissues.

The observation of a highly functional, non-exhausted T cell response in a persistent infection is significant, as retention of a robust CD8^+^ T cell function is rarely reported in chronic infection systems, especially after hundreds of days of infection. These data indicate that exhausted T cell responses and compromised immunity are not the only possible outcomes of persistent infection. The absence of exhaustion and exhausted T cell responses in chronic *T*. *cruzi* infection is supported by three distinct lines of evidence in this study: 1) the clear maintenance of on-going activation related to the persistent infection and the ability of parasite-specific T cells to be activated further, both in vivo and in vitro, characteristics often lost in systems with highly regulated T cells [49–51], 2) the demonstration of a requirement for both the presence of a fully potent CD8^+^ T cell compartment and for the homing of those T cells to sites of parasite persistence, and 3) the low to null expression of multiple markers associated with T cell exhaustion on *T*. *cruzi*–specific CD8^+^ T cells a year or more into the infection. We further show that preventing PD-1 or IL-10 regulatory pathways has no impact on parasite control, although, admittedly infection control is so effective (and thus parasite load so low) that detecting such an impact if it existed would be difficult. Nevertheless, when combined with previous studies from our group demonstrating that deleting or blocking of Treg action or TGF-β production also has no impact on parasite control in this infection [52, 53] we conclude that a compromised CD8^+^ T cell response is not responsible for *T*. *cruzi* persistence.

Why is it that persistent *T*. *cruzi* infection in this natural host system fails to exhaust the T cell response? One possibility is that the high efficiency of immune control in *T*. *cruzi* infection dramatically limits parasite load, and thus prevents, or limits the degree of chronic and continuous T cell stimulation. Very low parasite levels are characteristic of *T*. *cruzi* infection in nearly all hosts and indeed the inability to consistently detect parasites in blood or in tissues of chronically infected hosts is a major impediment to detection of infection and the assessment of treatment outcomes [54]. The role of antigen load in driving immune exhaustion has been well documented in a number of viral models [55, 56]. In HIV infection, early administration of anti-retroviral drugs significantly reduces viral levels and protects CD8^+^ T cells from the extreme antigen stimulation that drives HIV-specific T cell exhaustion [57, 58]. Similarly, during chronic HCV infection, a system also typically considered to exhibit relatively low antigen levels, CD8^+^ T cell exhaustion is observed primarily in patients with high viral titers [37]. Interestingly, patients with superior HCV control retain a pool of CD127+ antigen-specific memory CD8^+^ T cells during chronic infection, which serves as an indicator for improved control and limited T cell regulation [59]. A CD127+ Tcm population capable of antigen-independent survival is also evident during very chronic *T*. *cruzi* infections and after complete parasite clearance following benznidazole treatment [29, 60]. The maintenance of this memory population, and the requirement for the recruitment of new effector cells from this memory and/or the naïve T cell pool in order to maintain parasite control (Fig 4), suggests that low antigen levels, characteristic of *T*. *cruzi* infection, likely preserve CD8^+^ T cell function while maintaining pathogen control during chronic infection.

One could argue that while the anti-*T*. *cruzi* T cell response is qualitatively sound it may be quantitatively deficient. This seems unlikely since the immunodominant TSKB20-specific response alone represents between 5 and 10% of the total circulating CD8^+^ T cell compartment throughout the chronic infection (Fig 5A and [26, 29, 61]. Further, efforts to increase the strength of the CD8^+^ T cell response by immune enhancement [62] or by vaccination or reinfection [63] have failed to prevent parasite persistence.

Although we failed to observe significant T cell regulation or exhaustion in the infection models used in this study, it is likely that there are conditions in which compromised T cell function in *T*. *cruzi* infection could occur. Indeed, a subset of humans with *T*. *cruzi* infections lasting decades show hallmarks of terminal T cell differentiation, including inhibitory receptor expression [44, 64–68]. Interestingly children, who likely have shorter infection times relative to adults, retain more robust *T*. *cruzi*–specific T cell responses [65]. Likewise, we would expect that in a higher antigen system (e.g. high initial infection dose, a particularly virulent parasite strain, or host genetic conditions that result in suboptimal parasite control) that some level of immune regulation/exhaustion might become evident [69]. Indeed in B cell deficient mice, that fail to control acute *T*. *cruzi* and die with high parasite load, CD8^+^ T cell exhaustion accompanied by PD-1 and LAG3 expression is evident [69]. Thus, both the level of antigen exposure and the period over which that antigen is persistent may contribute to T cell exhaustion when either is in the extreme. With respect to *T*. *cruzi* infection, the predominant outcome appears to be that immune exhaustion, if it occurs, is the *result* of pathogen persistence, not the cause.

It remains unclear precisely how *T*. *cruzi* manages to persist despite the presence of highly functional CD8^+^ T cells (and other immune effectors) but this almost certainly is a result of very effective immune evasion mechanisms. Among these are 1) the relatively poor activation of innate responses due to the absence of potent pathogen associated molecular patterns (PAMPs) [70], 2) the focusing of the dominant CD8^+^ T cell response on a massive family of highly variant trans-sialidase molecules [26], 3) the delayed expression of these dominant CD8^+^ T cell targets on infected host cells [71], 4) the ability of *T*. *cruzi* to establish infection in many different tissue types, including ones with very low levels of class I MHC (e.g. muscle, [72–74]), and 5) the propensity of *T*. *cruzi* to go dormant in infected host cells [28]. Collectively, these factors would significantly impact the immune recognition of *T*. *cruzi*-infected cells and enable the survival of a nominal number of parasites, despite the presence of potent parasite-specific CD8^+^ T cells.

Here we have demonstrated that immunity to *T*. *cruzi* infection is long lasting and highly effective and that CD8^+^ T cell exhaustion is an unlikely explanation for long-term parasite persistence. Our study highlights the value of examining immune response at the host-pathogen interface in the actual tissues of persistence. This approach permitted the detection of a previously unappreciated population of activated, cytokine-producing, CD8^+^ T cells that tightly control *T*. *cruzi* levels. These findings further emphasize that immune exhaustion is not necessarily rapid nor the ultimate outcome of all persistent infections. Balancing pathogen control and the maintenance of the mechanisms that mediate that control, facilitates long-term host survival despite pathogen persistence. In *T*. *cruzi* infection, this is an effective, but imperfect balance since decades of infection may ultimately lead to clinical disease, as observed in a minority of chronically infected humans.

These findings also accentuate the challenge of designing immunological interventions for this, and possibly other persistent infections. Given the lack of evidence for immunoregulatory mechanisms in limiting pathogen control, it is unlikely that the blockade of checkpoint inhibitors which have proven effective in other systems [75–77] will be beneficial here and, in fact, may be deleterious [78]. Enhancing immune activation through increased antigen presentation also runs the risk of upsetting the balance and accelerating immune exhaustion [56, 79]. For *T*. *cruzi* infection, we might also take a lesson from persistent viral infections wherein driving down pathogen load with anti-viral drugs also has immunological benefits that contribute to long-term pathogen control and reduced pathology.

## Methods

### Mice and parasites

For *T*. *cruzi* infections, mice of 8–12 weeks old were infected via intraperitoneal (i.p.) injection of 10^3^ or 10^4^ trypomastigotes of the Brazil unless otherwise noted. In some experiments, the low virulence TCC strain or more highly virulent Colombiana strains were used, as indicated. The plasmid, pTRIX2-RE9h, a kind gift from Dr. John Kelly, London School of Hygiene and Tropical Medicine, London, UK [80], was used to generate Colombiana strain parasites that express red-shifted firefly luciferase for the indirect determination of parasite levels by quantification of luminescent signal. Parasites were maintained in culture using serial passage through Vero cells (American Type Culture Collection (ATCC)).

C57BL/6 mice (CD45.2+), B6.SJL (CD45.1+), B6.129S4-Ifng^tm3.1Lky^/J (IFNγ eYFP “GREAT”)[81], and B6.129P2-*Il10*^*tm1Cgn*^/J (IL-10 KO) mice were obtained from The Jackson Laboratory (Bar Harbor, ME), the National Cancer Institute (Frederick, MD) or were bred and maintained under specific pathogen-free conditions at the Coverdell Vivarium (University of Georgia, Athens, GA). PD-L1 KO mice were a kind gift from Dr. Arlene Sharp, Brigham and Women’s Hospital, Boston, MA. All mice were euthanized by CO_2_.

### Ethics statement

All animal use was performed in accordance with protocol A2014 09-017-R2 approved by the University of Georgia Institutional Animal Care and Use Committee. This protocol adhered to the animal welfare guidelines outlined in Guide for the Care and Use of Laboratory Animals.

### Lymphocyte isolation from peripheral tissues

In a small number of experiments, lymphocytes were isolated from peripheral tissues as previously described [82]. Most frequently, the following protocol was utilized with a few modifications [83]. Prior to tissue removal, mice were perfused by injection of 20 mL of 0.8% sodium citrate solution in PBS. Tissues were then minced and stirred in Hank’s balanced salt solution (Corning) with 1.25mM EDTA for 30 min at 37°C. This treatment was followed by incubation with 150U/mL collagenase (Gibco) in RPMI for at least 1h. The digested tissue was then filtered through a 70μM nylon cell strainer (BD Biosciences) and pelleted via centrifugation. The pellet was resuspended in 44% Percoll (GE Healthcare) then underlain with 67% Percoll in PBS. Following centrifugation at 600x g, cells were collected from the gradient interface and washed in RPMI.

### T cell phenotyping

CD8^+^ T cell phenotypes were determined by staining with the MHC class I tetramer TSKB20 (ANYKFTLV/K^b^) synthesized at the Tetramer Core Facility (Emory University, Atlanta, GA) and the following: CD8, CD44, KLRG1 (eBioscience), CD69, PD-1 (BD Pharmingen), and CD8 (Accurate Chemical). RBCs in single-cell suspensions of spleen cells were lysed in a hypotonic ammonium chloride solution and washed in staining buffer (2% BSA and 0.02% azide in PBS (PAB)). Cells were stained at 4°C, washed in PAB, and fixed in 2% formaldehyde. At least 100,000 lymphocytes were acquired using a HyperCyAn or CyAn flow cytometer (DakoCytomation) and analyzed with FlowJo software (Tree Star).

### Standard T cell stimulation and intracellular staining

Lymphocytes isolated from spleen or peripheral tissue were stimulated with 1.5 μg of plate-bound anti-mouse CD3ε (eBioscience) for 5h at 37°C in the presence of Golgi Plug (BD Pharmingen). Cells were surface stained, washed, fixed and permeabilized, and intracellular cytokine staining was performed to detect IFNγ (eBioscience) and TNFα (BD Pharmingen) using a Cytofix/Cytoperm kit (BD Pharmingen) in accordance with the manufacturer’s instructions.

### Direct intracellular cytokine staining and in vivo activation

For direct intracellular staining (dICS) [22], mice were injected with 0.25 mg of brefeldin A (Sigma) in PBS and 6-8h later, tissues were collected and processed as described above with the addition of brefeldin (10 μg/mL) to isolation solutions. For intracellular granzyme B (BD Pharmingen) staining, cells were stained directly *ex vivo* without stimulation using the Cytofix/Cytoperm kit (BD Pharmingen) following surface staining. For *in vivo* stimulation with synthetic peptides [84], mice with injected i.p. with 100 μg of TSKB20 (ANYKFTLV) or control SIINFEKL peptide (GenScript) (simultaneously with brefeldin A administration). Cells were then processed using the typical dICS protocol.

### *In vivo* cytolysis assay

Spleens from naïve mice were collected and prepared as described above. Cells were then with pulsed with 10 μM of TSKB20 peptide (Genescript) for 1h. Peptide-pulsed splenocytes were then labeled with 5 μM CFSE and mixed in a 1:1 ratio with unpulsed cells labeled with 2.5 uM CFSE, prior to IV delivery to chronically (>250 dpi) infected mice. Lymphocytes were recovered from spleen and muscle ~18 hours later, as described above and were assessed.

### *In vivo* BrdU incorporation

A bromodeoxyuridine (BrdU) (Sigma) solution (0.8 mg/mL) was prepared fresh every 2d and administered *ad libitum* in drinking water to naïve and chronically infected mice for 21d. Lymphocytes were isolated from skeletal muscle and spleen as described above. Staining was then performed with anti-BrdU antibody in accordance with manufacturer instructions in the BrdU Flow Kit (BD Pharmingen).

### Treatments

Blocking or depleting monoclonal antibodies (mAb) were administered by i.p. injection every third day for 30 days with the exception of anti-CD8a which was administered every other day for 21 days. The clone, specificity, and amount administered with each treatment were as follows: anti-IL-10R (1B1.3A) 250 μg; anti-CD8a (YTS 169.4) 200 μg; anti-VLA-4 (PS/2) 150 μg; anti-PD-L1 (MIH5) 200 μg. The anti-CD8a Ab used for detection by flow cytometry is a distinct clone from the depleting Ab used.

Mice were treated with the trypanocidal drug benznidazole (Roche) to reduce the parasite load in chronically infected mice. The compound was administered orally (100 mg/kg body weight) for 21 days.

### Adoptive transfer of CD8^+^ T cells

For transfers, spleens from chronically *T*. *cruzi*-infected mice were homogenized with frosted glass slides (Fisher Scientific) in a hypotonic ammonium chloride RBC lysis buffer and washed in RPMI with 10% FBS. CD8^+^ T cells were negatively selected through magnetic sorting with a CD8a^+^ T cell isolation kit (Miltenyi). CD8^+^ cells were transferred i.v. into infection-matched congenic mice, and recipients were terminated at various days post transfer to analyze donor cell populations in recipient tissues.

### Quantitation of parasite burden

Parasite equivalents in tissue were determined as previously described [85]. Briefly, tissue was collected from mice and finely minced. Samples were incubated at 55 °C in SDS-proteinase K lysis buffer. DNA was extracted twice with phenol:chloroform:isoamyl alcohol (25:24:1), precipitated with 100% ethanol, and resuspended in nuclease free water. PCR reactions contained iQ SYBR Green Supermix (Biorad) and primers specific for *T*. *cruzi* or mouse genomic DNA. Samples were analyzed on an iCycler (Biorad) and *T*. *cruzi* equivalents were calculated as the ratio of *T*. *cruzi* satellite DNA divided by the quantity of mouse TNFα DNA in each sample.

Parasite burden in select tissues and organs was assessed via *ex vivo* imaging following infection with luciferase expressing parasites. Mice were perfused with 25 mL of D-luciferin (Gold Bio) at 0.3 mg/mL in PBS via the heart. Excised tissues were transferred to a culture dish, soaked in 0.3 mg/ml of D-luciferin in PBS, and then imaged using an IVIS Lumina II system (Xenogen). Exposure time was 5 minutes.

### Histology

Skeletal muscle was obtained from *T*. *cruzi*-infected mice and controls, fixed in 10% buffered formalin and embedded in paraffin. Five-micron thick sections were obtained and stained with hematoxylin-eosin. Inflammation was evaluated qualitatively according to the presence or absence of myocyte necrosis and infiltrates according to distribution (focal, confluent or diffuse) and extent of inflammatory cells present. Images of tissue sections were taken with an OLYMPUS DP70 digital camera on an OLYMPUS BX60 microscope.

### Statistical analysis

Statistical significance was calculated using a two-tailed Student’s t-test or Mann-Whitney test. * indicates values (mean+/- SEM) that are significantly different between specified groups (* P ≤ 0.05, ** P ≤ 0.01,***P ≤ .001).

## Supporting information

S1 Fig CD69 expression by CD8^+^ T cells is related to parasite antigen level during chronic infection. (A) Recently-activated CD8^+^ T cells are preferentially found at sites of parasite persistence such as heart and fat. Representative flow plots show surface expression of CD69 for cells isolated from indicated tissue during chronic (~230 dpi) *T*. *cruzi* infection. Plots gated on CD8^+^ T cells. Numbers in upper right indicate the percentage of CD69+ TSKB20+ cells; numbers in lower right indicate the percentage of TSKB20- cells expressing CD69. Data are representative of at least 2 independent experiments with n = 4–6 and depict mean+ SEM. * indicates percentage levels that are significantly different (* P ≤ 0.05, ** P ≤ 0.01, ***P ≤ 0.001) between specified groups.
(TIF)

S2 Fig Inhibitory receptor expression has a minor impact on CD8+ T cells during chronic infection. (A) Histograms display LAG-3 and TIM-3 expression by muscle and spleen CD44+ CD8^+^ T cells at >500 dpi from infected (magenta), isotype control (gray), naïve (blue) mice. The percentage of inhibitory receptor positive cells observed is described graphically for the total CD8^+^ and (B) TSKB20+ populations.
(TIF)

S3 Fig PD-L1 blockade does not enhance CD8^+^ T cell response to *ex vivo* stimulation. CD8^+^ T cells from chronically infected mice treated for 30 days with PD-L1 blocking antibody were stimulated for 5 hours with anti-mouse CD3ε. (A) The frequency of IFNγ+ (white), TNFα+ (black), and IFNγ+ and TNFα+ CD8^+^ T cells in the muscle (left) and spleen (right) is not increased by PD-L1 blockade.
(TIF)

S4 Fig IL-10 is not a major factor controlling CD8^+^ T cells in *T*. *cruzi* infection. (A) IL-10 KO and WT mice exhibit similar parasite burden. Parasite load in skeletal muscle of IL-10 KO and WT mice during acute (30 dpi) *T*. *cruzi* infection was assessed by real-time PCR. (B) IL-10 KO mice cannot control the inflammatory response to *T*. *cruzi*. H&E sections of skeletal muscle from acutely infected IL-KO and WT mice. (C) Interrupting IL-10 signaling does not promote improved clearance of *T*. *cruzi*. Parasite loads in skeletal muscle of chronically *T*. *cruzi*-infected mice receiving anti-IL-10R Ab or rat IgG are plotted. The dashed line represents the limit of detection for quantitative real-time PCR. Bars show mean, which were not statistically different by Mann-Whitney test. Similar results were obtained in a repeat of each experiment.
(TIF)

## References

[1] Feldman AM, McNamara D. Myocarditis. The New England journal of medicine. 2000;343(19):1388–98. 10.1056/NEJM200011093431908 . 11070105 10.1056/NEJM200011093431908

[2] Schofield CJ, Jannin J, Salvatella R. The future of Chagas disease control. Trends in parasitology. 2006;22(12):583–8. Epub 2006/10/20. 10.1016/j.pt.2006.09.011 . 17049308 10.1016/j.pt.2006.09.011

[3] Bern C, Montgomery SP, Herwaldt BL, Rassi A Jr., Marin-Neto JA, Dantas RO, et al Evaluation and treatment of chagas disease in the United States: a systematic review. Jama. 2007;298(18):2171–81. 10.1001/jama.298.18.2171 . 18000201 10.1001/jama.298.18.2171

[4] Harty JT, Bevan MJ. Responses of CD8(+) T cells to intracellular bacteria. Current opinion in immunology. 1999;11(1):89–93. . 10047532 10.1016/s0952-7915(99)80016-8

[5] Wong P, Pamer EG. CD8 T cell responses to infectious pathogens. Annu Rev Immunol. 2003;21:29–70. 10.1146/annurev.immunol.21.120601.141114 . 12414723 10.1146/annurev.immunol.21.120601.141114

[6] Wherry EJ, Blattman JN, Murali-Krishna K, van der Most R, Ahmed R. Viral persistence alters CD8 T-cell immunodominance and tissue distribution and results in distinct stages of functional impairment. Journal of virology. 2003;77(8):4911–27. Epub 2003/03/29. 10.1128/JVI.77.8.4911-4927.2003 . 12663797 10.1128/JVI.77.8.4911-4927.2003 PMC152117

[7] Fuller MJ, Khanolkar A, Tebo AE, Zajac AJ. Maintenance, loss, and resurgence of T cell responses during acute, protracted, and chronic viral infections. J Immunol. 2004;172(7):4204–14. Epub 2004/03/23. . 15034033 10.4049/jimmunol.172.7.4204

[8] Mueller SN, Ahmed R. High antigen levels are the cause of T cell exhaustion during chronic viral infection. Proceedings of the National Academy of Sciences of the United States of America. 2009;106(21):8623–8. 10.1073/pnas.0809818106 . 19433785 10.1073/pnas.0809818106 PMC2688997

[9] Wherry EJ, Kurachi M. Molecular and cellular insights into T cell exhaustion. Nature Reviews Immunology. 2015;15(8):486–99. 10.1038/nri3862 26205583 10.1038/nri3862 PMC4889009

[10] Penaloza-MacMaster P, Kamphorst AO, Wieland A, Araki K, Iyer SS, West EE, et al Interplay between regulatory T cells and PD-1 in modulating T cell exhaustion and viral control during chronic LCMV infection. The Journal of experimental medicine. 2014;211(9):1905–18. Epub 2014/08/13. 10.1084/jem.20132577 . 25113973 10.1084/jem.20132577 PMC4144726

[11] Brooks DG, Trifilo MJ, Edelmann KH, Teyton L, McGavern DB, Oldstone MB. Interleukin-10 determines viral clearance or persistence in vivo. Nature medicine. 2006;12(11):1301–9. Epub 2006/10/17. 10.1038/nm1492 . 17041596 10.1038/nm1492 PMC2535582

[12] Ejrnaes M, Filippi CM, Martinic MM, Ling EM, Togher LM, Crotty S, et al Resolution of a chronic viral infection after interleukin-10 receptor blockade. The Journal of experimental medicine. 2006;203(11):2461–72. Epub 2006/10/13. 10.1084/jem.20061462 . 17030951 10.1084/jem.20061462 PMC2118120

[13] Tinoco R, Alcalde V, Yang Y, Sauer K, Zuniga EI. Cell-intrinsic transforming growth factor-beta signaling mediates virus-specific CD8+ T cell deletion and viral persistence in vivo. Immunity. 2009;31(1):145–57. Epub 2009/07/17. 10.1016/j.immuni.2009.06.015 . 19604493 10.1016/j.immuni.2009.06.015 PMC3039716

[14] Tarleton RL. Depletion of CD8+ T cells increases susceptibility and reverses vaccine-induced immunity in mice infected with Trypanosoma cruzi. J Immunol. 1990;144(2):717–24. Epub 1990/01/15. . 2104903

[15] Tarleton RL, Koller BH, Latour A, Postan M. Susceptibility of beta 2-microglobulin-deficient mice to Trypanosoma cruzi infection. Nature. 1992;356(6367):338–40. 10.1038/356338a0 1549177 10.1038/356338a0

[16] Tarleton RL, Sun J, Zhang L, Postan M. Depletion of T-cell subpopulations results in exacerbation of myocarditis and parasitism in experimental Chagas’ disease. Infection and immunity. 1994;62(5):1820–9. . 8168945 10.1128/iai.62.5.1820-1829.1994 PMC186416

[17] Perez-Anton E, Egui A, Thomas MC, Puerta CJ, Gonzalez JM, Cuellar A, et al Impact of benznidazole treatment on the functional response of Trypanosoma cruzi antigen-specific CD4+CD8+ T cells in chronic Chagas disease patients. PLoS neglected tropical diseases. 2018;12(5):e0006480 10.1371/journal.pntd.0006480 . 29750791 10.1371/journal.pntd.0006480 PMC5965897

[18] Gutierrez FR, Mariano FS, Oliveira CJ, Pavanelli WR, Guedes PM, Silva GK, et al Regulation of Trypanosoma cruzi-induced myocarditis by programmed death cell receptor 1. Infection and immunity. 2011;79(5):1873–81. Epub 2011/03/02. 10.1128/IAI.01047-10 . 21357717 10.1128/IAI.01047-10 PMC3088162

[19] Joshi T, Rodriguez S, Perovic V, Cockburn IA, Stager S. B7-H1 blockade increases survival of dysfunctional CD8(+) T cells and confers protection against Leishmania donovani infections. PLoS pathogens. 2009;5(5):e1000431 Epub 2009/05/14. 10.1371/journal.ppat.1000431 . 19436710 10.1371/journal.ppat.1000431 PMC2674929

[20] Bhadra R, Gigley JP, Khan IA. PD-1-mediated attrition of polyfunctional memory CD8+ T cells in chronic toxoplasma infection. The Journal of infectious diseases. 2012;206(1):125–34. Epub 2012/04/28. 10.1093/infdis/jis304 . 22539813 10.1093/infdis/jis304 PMC3415930

[21] Horne-Debets JM, Faleiro R, Karunarathne DS, Liu XQ, Lineburg KE, Poh CM, et al PD-1 dependent exhaustion of CD8+ T cells drives chronic malaria. Cell reports. 2013;5(5):1204–13. Epub 2013/12/10. 10.1016/j.celrep.2013.11.002 . 24316071 10.1016/j.celrep.2013.11.002

[22] Liu F, Whitton JL. Cutting edge: re-evaluating the in vivo cytokine responses of CD8+ T cells during primary and secondary viral infections. J Immunol. 2005;174(10):5936–40. Epub 2005/05/10. . 15879085 10.4049/jimmunol.174.10.5936

[23] Corbin GA, Harty JT. T cells undergo rapid ON/OFF but not ON/OFF/ON cycling of cytokine production in response to antigen. J Immunol. 2005;174(2):718–26. Epub 2005/01/07. . 15634891 10.4049/jimmunol.174.2.718

[24] Engelhardt KR, Richter K, Baur K, Staeheli P, Hausmann J. The functional avidity of virus-specific CD8+ T cells is downmodulated in Borna disease virus-induced immunopathology of the central nervous system. European journal of immunology. 2005;35(2):487–97. 10.1002/eji.200425232 15627979 10.1002/eji.200425232

[25] Zhang L, Tarleton RL. Parasite persistence correlates with disease severity and localization in chronic Chagas’ disease. The Journal of infectious diseases. 1999;180(2):480–6. 10.1086/314889 10395865 10.1086/314889

[26] Martin DL, Weatherly DB, Laucella SA, Cabinian MA, Crim MT, Sullivan S, et al CD8+ T-Cell responses to Trypanosoma cruzi are highly focused on strain-variant trans-sialidase epitopes. PLoS pathogens. 2006;2(8):e77 10.1371/journal.ppat.0020077 . 16879036 10.1371/journal.ppat.0020077 PMC1526708

[27] Gebhardt T, Palendira U, Tscharke DC, Bedoui S. Tissue-resident memory T cells in tissue homeostasis, persistent infection, and cancer surveillance. Immunological reviews. 2018;283(1):54–76. 10.1111/imr.12650 . 29664571 10.1111/imr.12650

[28] Sanchez-Valdez FJ, Padilla A, Wang W, Orr D, Tarleton RL. Spontaneous dormancy protects Trypanosoma cruzi during extended drug exposure. eLife. 2018;7 10.7554/eLife.34039 . 29578409 10.7554/eLife.34039 PMC5906098

[29] Bustamante JM, Bixby LM, Tarleton RL. Drug-induced cure drives conversion to a stable and protective CD8+ T central memory response in chronic Chagas disease. Nat Med. 2008;14(5):542–50. 10.1038/nm1744 . 18425131 10.1038/nm1744 PMC3074975

[30] Siegelman MH, Stanescu D, Estess P. The CD44-initiated pathway of T-cell extravasation uses VLA-4 but not LFA-1 for firm adhesion. Journal of Clinical Investigation. 2000;105(5):683–91. 10.1172/JCI8692 10712440 10.1172/JCI8692 PMC292454

[31] Day CL, Abrahams DA, Lerumo L, Janse van Rensburg E, Stone L, O’Rie T, et al Functional capacity of Mycobacterium tuberculosis-specific T cell responses in humans is associated with mycobacterial load. J Immunol. 2011;187(5):2222–32. 10.4049/jimmunol.1101122 . 21775682 10.4049/jimmunol.1101122 PMC3159795

[32] Jeong YH, Jeon BY, Gu SH, Cho SN, Shin SJ, Chang J, et al Differentiation of antigen-specific T cells with limited functional capacity during Mycobacterium tuberculosis infection. Infection and immunity. 2014;82(1):132–9. 10.1128/IAI.00480-13 . 24126533 10.1128/IAI.00480-13 PMC3911842

[33] Buggert M, Tauriainen J, Yamamoto T, Frederiksen J, Ivarsson MA, Michaelsson J, et al T-bet and Eomes are differentially linked to the exhausted phenotype of CD8+ T cells in HIV infection. PLoS pathogens. 2014;10(7):e1004251 10.1371/journal.ppat.1004251 . 25032686 10.1371/journal.ppat.1004251 PMC4102564

[34] Banga R, Procopio FA, Noto A, Pollakis G, Cavassini M, Ohmiti K, et al PD-1 and follicular helper T cells are responsible for persistent HIV-1 transcription in treated aviremic individuals. Nature medicine. 2016 10.1038/nm.4113 . 27239760 10.1038/nm.4113

[35] Tzeng HT, Tsai HF, Liao HJ, Lin YJ, Chen L, Chen PJ, et al PD-1 blockage reverses immune dysfunction and hepatitis B viral persistence in a mouse animal model. PloS one. 2012;7(6):e39179 10.1371/journal.pone.0039179 . 22761734 10.1371/journal.pone.0039179 PMC3382233

[36] Sauce D, Almeida JR, Larsen M, Haro L, Autran B, Freeman GJ, et al PD-1 expression on human CD8 T cells depends on both state of differentiation and activation status. AIDS. 2007;21(15):2005–13. 10.1097/QAD.0b013e3282eee548 . 17885290 10.1097/QAD.0b013e3282eee548

[37] Bengsch B, Seigel B, Ruhl M, Timm J, Kuntz M, Blum HE, et al Coexpression of PD-1, 2B4, CD160 and KLRG1 on exhausted HCV-specific CD8+ T cells is linked to antigen recognition and T cell differentiation. PLoS pathogens. 2010;6(6):e1000947 10.1371/journal.ppat.1000947 . 20548953 10.1371/journal.ppat.1000947 PMC2883597

[38] Brooks DG, Lee AM, Elsaesser H, McGavern DB, Oldstone MB. IL-10 blockade facilitates DNA vaccine-induced T cell responses and enhances clearance of persistent virus infection. The Journal of experimental medicine. 2008;205(3):533–41. Epub 2008/03/12. 10.1084/jem.20071948 . 18332180 10.1084/jem.20071948 PMC2275377

[39] Beamer GL, Flaherty DK, Assogba BD, Stromberg P, Gonzalez-Juarrero M, de Waal Malefyt R, et al Interleukin-10 promotes Mycobacterium tuberculosis disease progression in CBA/J mice. J Immunol. 2008;181(8):5545–50. Epub 2008/10/04. . 18832712 10.4049/jimmunol.181.8.5545 PMC2728584

[40] Bertocchi GL, Vigliano CA, Lococo BG, Petti MA, Viotti RJ. Clinical characteristics and outcome of 107 adult patients with chronic Chagas disease and parasitological cure criteria. Transactions of the Royal Society of Tropical Medicine and Hygiene. 2013;107(6):372–6. 10.1093/trstmh/trt029 . 23612468 10.1093/trstmh/trt029

[41] Francolino SS, Antunes AF, Talice R, Rosa R, Selanikio J, de Rezende JM, et al New evidence of spontaneous cure in human Chagas’ disease. Revista da Sociedade Brasileira de Medicina Tropical. 2003;36(1):103–7. . 12715069 10.1590/s0037-86822003000100014

[42] Dias JC D E, Martins-Filho OA, Vitelli-Avelar D, Correia D, Lages E, Prata A. Further evidence of spontaneous cure in human Chagas disease. Rev Soc Bras Med Trop. 2008;41:505–6. 19009195 10.1590/s0037-86822008000500014

[43] Tarleton R. The role of immunology in combating Trypanosoma cruzi infection and Chagas disease. Rev Esp Salud Publica. 2013;86:33–9.

[44] Arguello RJ, Albareda MC, Alvarez MG, Bertocchi G, Armenti AH, Vigliano C, et al Inhibitory receptors are expressed by Trypanosoma cruzi-specific effector T cells and in hearts of subjects with chronic Chagas disease. PloS one. 2012;7(5):e35966 Epub 2012/05/11. 10.1371/journal.pone.0035966 . 22574131 10.1371/journal.pone.0035966 PMC3344843

[45] Dutra WO, Menezes CA, Magalhaes LM, Gollob KJ. Immunoregulatory networks in human Chagas disease. Parasite Immunol. 2014;36(8):377–87. 10.1111/pim.12107 . 24611805 10.1111/pim.12107 PMC4143493

[46] Butler NS, Moebius J, Pewe LL, Traore B, Doumbo OK, Tygrett LT, et al Therapeutic blockade of PD-L1 and LAG-3 rapidly clears established blood-stage Plasmodium infection. Nature immunology. 2012;13(2):188–95. 10.1038/ni.2180 . 22157630 10.1038/ni.2180 PMC3262959

[47] Zajac AJ, Blattman JN, Murali-Krishna K, Sourdive DJD, Suresh M, Altman JD, et al Viral immune evasion due to persistence of activated T cells without effector function. Journal of Experimental Medicine. 1998;188(12):2205–13. 10.1084/jem.188.12.2205 9858507 10.1084/jem.188.12.2205 PMC2212420

[48] Bhadra R, Gigley JP, Weiss LM, Khan IA. Control of Toxoplasma reactivation by rescue of dysfunctional CD8+ T-cell response via PD-1-PDL-1 blockade. Proceedings of the National Academy of Sciences of the United States of America. 2011;108(22):9196–201. 10.1073/pnas.1015298108 . 21576466 10.1073/pnas.1015298108 PMC3107287

[49] Kahan SM, Wherry EJ, Zajac AJ. T cell exhaustion during persistent viral infections. Virology. 2015;479–480:180–93. 10.1016/j.virol.2014.12.033 . 25620767 10.1016/j.virol.2014.12.033 PMC4424083

[50] Wherry EJ, Kurachi M. Molecular and cellular insights into T cell exhaustion. Nature reviews Immunology. 2015;15(8):486–99. 10.1038/nri3862 . 26205583 10.1038/nri3862 PMC4889009

[51] Gupta PK, Godec J, Wolski D, Adland E, Yates K, Pauken KE, et al CD39 Expression Identifies Terminally Exhausted CD8+ T Cells. PLoS pathogens. 2015;11(10):e1005177 10.1371/journal.ppat.1005177 . 26485519 10.1371/journal.ppat.1005177 PMC4618999

[52] Kotner J, Tarleton R. Endogenous CD4(+) CD25(+) regulatory T cells have a limited role in the control of Trypanosoma cruzi infection in mice. Infection and immunity. 2007;75(2):861–9. 10.1128/IAI.01500-06 . 17101658 10.1128/IAI.01500-06 PMC1828478

[53] Martin DL, Postan M, Lucas P, Gress R, Tarleton RL. TGF-beta regulates pathology but not tissue CD8+ T cell dysfunction during experimental Trypanosoma cruzi infection. European journal of immunology. 2007;37(10):2764–71. 10.1002/eji.200737033 . 17823982 10.1002/eji.200737033

[54] Tarleton RL, Reithinger R, Urbina JA, Kitron U, Gurtler RE. The challenges of Chagas Disease—grim outlook or glimmer of hope. PLoS medicine. 2007;4(12):e332 10.1371/journal.pmed.0040332 . 18162039 10.1371/journal.pmed.0040332 PMC2222930

[55] Bucks CM, Norton JA, Boesteanu AC, Mueller YM, Katsikis PD. Chronic antigen stimulation alone is sufficient to drive CD8+ T cell exhaustion. J Immunol. 2009;182(11):6697–708. 10.4049/jimmunol.0800997 . 19454664 10.4049/jimmunol.0800997 PMC2923544

[56] Richter K, Brocker T, Oxenius A. Antigen amount dictates CD8+ T-cell exhaustion during chronic viral infection irrespective of the type of antigen presenting cell. European journal of immunology. 2012;42(9):2290–304. 10.1002/eji.201142275 . 22653665 10.1002/eji.201142275

[57] Streeck H, Brumme ZL, Anastario M, Cohen KW, Jolin JS, Meier A, et al Antigen load and viral sequence diversification determine the functional profile of HIV-1-specific CD8+ T cells. PLoS medicine. 2008;5(5):e100 10.1371/journal.pmed.0050100 . 18462013 10.1371/journal.pmed.0050100 PMC2365971

[58] Petrovas C, Casazza JP, Brenchley JM, Price DA, Gostick E, Adams WC, et al PD-1 is a regulator of virus-specific CD8+ T cell survival in HIV infection. J Exp Med. 2006;203(10):2281–92. 10.1084/jem.20061496 . 16954372 10.1084/jem.20061496 PMC2118095

[59] Wherry EJ, Barber DL, Kaech SM, Blattman JN, Ahmed R. Antigen-independent memory CD8 T cells do not develop during chronic viral infection. Proceedings of the National Academy of Sciences of the United States of America. 2004;101(45):16004–9. 10.1073/pnas.0407192101 . 15505208 10.1073/pnas.0407192101 PMC524220

[60] Bixby LM, Tarleton RL. Stable CD8(+) T cell memory during persistent Trypanosoma cruzi infection. Journal of Immunology. 2008;181(4):2644–50. 10.4049/jimmunol.181.4.2644 PMC2735072 18684955

[61] Bixby LM, Tarleton RL. Stable CD8+ T cell memory during persistent Trypanosoma cruzi infection. Journal of immunology. 2008;181(4):2644–50. . 18684955 10.4049/jimmunol.181.4.2644 PMC2735072

[62] Kurup SP, Tarleton RL. Perpetual expression of PAMPs necessary for optimal immune control and clearance of a persistent pathogen. Nature communications. 2013;4:2616 Epub 2013/10/24. 10.1038/ncomms3616 . 24149620 10.1038/ncomms3616 PMC4161029

[63] Bustamante J, Tarleton R. Reaching for the Holy Grail: insights from infection/cure models on the prospects for vaccines for Trypanosoma cruzi infection. Memorias do Instituto Oswaldo Cruz. 2015;110(3):445–51. 10.1590/0074-02760140440 . 25946159 10.1590/0074-02760140440 PMC4489482

[64] Laucella SA, Postan M, Martin D, Hubby Fralish B, Albareda MC, Alvarez MG, et al Frequency of interferon- gamma -producing T cells specific for Trypanosoma cruzi inversely correlates with disease severity in chronic human Chagas disease. The Journal of infectious diseases. 2004;189(5):909–18. 10.1086/381682 . 14976609 10.1086/381682

[65] Albareda MC, De Rissio AM, Tomas G, Serjan A, Alvarez MG, Viotti R, et al Polyfunctional T cell responses in children in early stages of chronic Trypanosoma cruzi infection contrast with monofunctional responses of long-term infected adults. PLoS neglected tropical diseases. 2013;7(12):e2575 10.1371/journal.pntd.0002575 . 24349591 10.1371/journal.pntd.0002575 PMC3861186

[66] Lasso P, Mateus J, Pavia P, Rosas F, Roa N, Thomas MC, et al Inhibitory Receptor Expression on CD8+ T Cells Is Linked to Functional Responses against Trypanosoma cruzi Antigens in Chronic Chagasic Patients. J Immunol. 2015;195(8):3748–58. Epub 2015/09/20. 10.4049/jimmunol.1500459 . 26385520 10.4049/jimmunol.1500459

[67] Mateus J, Lasso P, Pavia P, Rosas F, Roa N, Valencia-Hernandez CA, et al Low frequency of circulating CD8+ T stem cell memory cells in chronic chagasic patients with severe forms of the disease. PLoS neglected tropical diseases. 2015;9(1):e3432 Epub 2015/01/09. 10.1371/journal.pntd.0003432 . 25569149 10.1371/journal.pntd.0003432 PMC4287481

[68] Albareda MC, Laucella SA, Alvarez MG, Armenti AH, Bertochi G, Tarleton RL, et al Trypanosoma cruzi modulates the profile of memory CD8+ T cells in chronic Chagas’ disease patients. Int Immunol. 2006;18(3):465–71. 10.1093/intimm/dxh387 . 16431876 10.1093/intimm/dxh387

[69] Sullivan NL, Eickhoff CS, Sagartz J, Hoft DF. Deficiency of Antigen-Specific B Cells Results in Decreased Trypanosoma cruzi Systemic but Not Mucosal Immunity Due to CD8 T Cell Exhaustion. Journal of immunology. 2015;194(4):1806–18. 10.4049/jimmunol.1303163 . 25595788 10.4049/jimmunol.1303163 PMC4324165

[70] Padilla AM, Simpson LJ, Tarleton RL. Insufficient TLR activation contributes to the slow development of CD8+ T cell responses in Trypanosoma cruzi infection. Journal of immunology. 2009;183(2):1245–52. 10.4049/jimmunol.0901178 . 19553540 10.4049/jimmunol.0901178

[71] Kurup SP, Tarleton RL. The Trypanosoma cruzi Flagellum Is Discarded via Asymmetric Cell Division following Invasion and Provides Early Targets for Protective CD8(+) T Cells. Cell host & microbe. 2014;16(4):439–49. 10.1016/j.chom.2014.09.003 . 25299330 10.1016/j.chom.2014.09.003 PMC4194031

[72] Frontera WR, Ochala J. Skeletal muscle: a brief review of structure and function. Calcif Tissue Int. 2015;96(3):183–95. 10.1007/s00223-014-9915-y . 25294644 10.1007/s00223-014-9915-y

[73] Daar AS, Fuggle SV, Fabre JW, Ting A, Morris PJ. The detailed distribution of HLA-A, B, C antigens in normal human organs. Transplantation. 1984;38(3):287–92. . 6591601 10.1097/00007890-198409000-00018

[74] Melo RC, Brener Z. Tissue tropism of different Trypanosoma cruzi strains. The Journal of parasitology. 1978;64(3):475–82. Epub 1978/06/01. . 96243

[75] Barber DL, Wherry EJ, Masopust D, Zhu B, Allison JP, Sharpe AH, et al Restoring function in exhausted CD8 T cells during chronic viral infection. Nature. 2006;439(7077):682–7. 10.1038/nature04444 . 16382236 10.1038/nature04444

[76] West EE, Jin HT, Rasheed AU, Penaloza-Macmaster P, Ha SJ, Tan WG, et al PD-L1 blockade synergizes with IL-2 therapy in reinvigorating exhausted T cells. The Journal of clinical investigation. 2013;123(6):2604–15. Epub 2013/05/17. 10.1172/JCI67008 . 23676462 10.1172/JCI67008 PMC3668811

[77] Jin HT, Anderson AC, Tan WG, West EE, Ha SJ, Araki K, et al Cooperation of Tim-3 and PD-1 in CD8 T-cell exhaustion during chronic viral infection. Proceedings of the National Academy of Sciences of the United States of America. 2010;107(33):14733–8. 10.1073/pnas.1009731107 . 20679213 10.1073/pnas.1009731107 PMC2930455

[78] Gutierrez FRS, Mariano FS, Oliveira CJF, Pavanelli WR, Guedes PMM, Silva GK, et al Regulation of Trypanosoma cruzi-Induced Myocarditis by Programmed Death Cell Receptor. Infection and immunity. 2011;79(5):1873–81. 10.1128/IAI.01047-10 21357717 10.1128/IAI.01047-10 PMC3088162

[79] Utzschneider DT, Alfei F, Roelli P, Barras D, Chennupati V, Darbre S, et al High antigen levels induce an exhausted phenotype in a chronic infection without impairing T cell expansion and survival. The Journal of experimental medicine. 2016 10.1084/jem.20150598 . 27455951 10.1084/jem.20150598 PMC4995073

[80] Lewis MD, Fortes Francisco A, Taylor MC, Burrell-Saward H, McLatchie AP, Miles MA, et al Bioluminescence imaging of chronic Trypanosoma cruzi infections reveals tissue-specific parasite dynamics and heart disease in the absence of locally persistent infection. Cellular microbiology. 2014;16(9):1285–300. 10.1111/cmi.12297 . 24712539 10.1111/cmi.12297 PMC4190689

[81] Reinhardt RL, Liang HE, Locksley RM. Cytokine-secreting follicular T cells shape the antibody repertoire. Nature immunology. 2009;10(4):385–93. Epub 2009/03/03. 10.1038/ni.1715 . 19252490 10.1038/ni.1715 PMC2714053

[82] Leavey JK, Tarleton RL. Cutting edge: dysfunctional CD8+ T cells reside in nonlymphoid tissues during chronic Trypanosoma cruzi infection. J Immunol. 2003;170(5):2264–8. Epub 2003/02/21. . 12594245 10.4049/jimmunol.170.5.2264

[83] Masopust D, Vezys V, Marzo AL, Lefrancois L. Preferential localization of effector memory cells in nonlymphoid tissue. Science. 2001;291(5512):2413–7. Epub 2001/03/27. 10.1126/science.1058867 . 11264538 10.1126/science.1058867

[84] Bold TD, Banaei N, Wolf AJ, Ernst JD. Suboptimal activation of antigen-specific CD4+ effector cells enables persistence of M. tuberculosis in vivo. PLoS pathogens. 2011;7(5):e1002063 Epub 2011/06/04. 10.1371/journal.ppat.1002063 . 21637811 10.1371/journal.ppat.1002063 PMC3102708

[85] Cummings KL, Tarleton RL. Rapid quantitation of Trypanosoma cruzi in host tissue by real-time PCR. Molecular and biochemical parasitology. 2003;129(1):53–9. . 12798506 10.1016/s0166-6851(03)00093-8

[86] Sanchez-Valdez FJ, Perez Brandan C, Ramirez G, Uncos AD, Zago MP, Cimino RO, et al A monoallelic deletion of the TcCRT gene increases the attenuation of a cultured Trypanosoma cruzi strain, protecting against an in vivo virulent challenge. PLoS neglected tropical diseases. 2014;8(2):e2696 10.1371/journal.pntd.0002696 . 24551259 10.1371/journal.pntd.0002696 PMC3923724

