# Supplementary figures and images for: Highly competent, non-exhausted CD8+ T cells continue to tightly control pathogen load throughout chronic Trypanosoma cruzi infection
Source: PLoS Pathog. 2018 Nov 12;14(11):e1007410. doi: 10.1371/journal.ppat.1007410 (PMC6258465; doi:10.1371/journal.ppat.1007410)

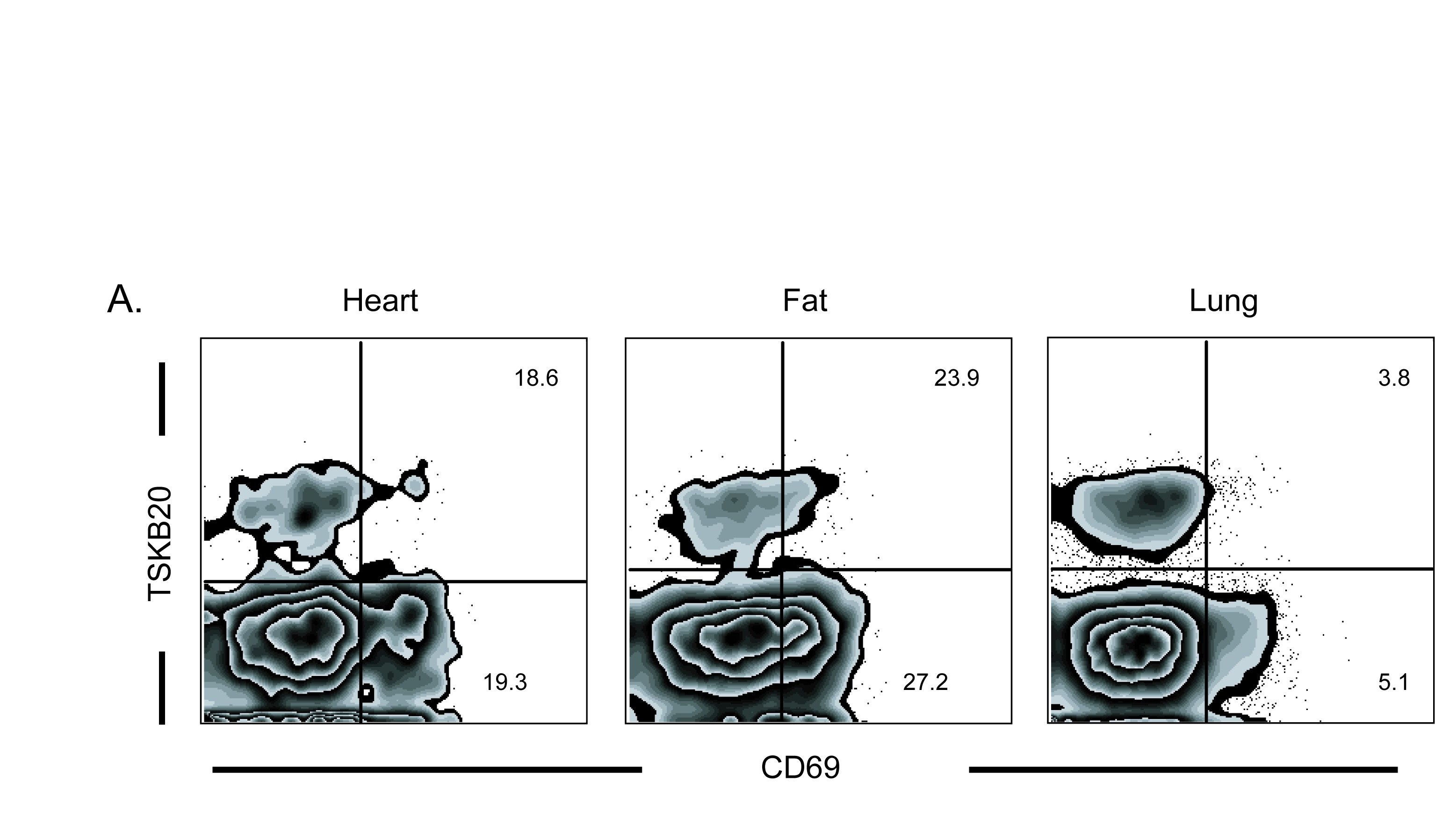

Supplement: S1 Fig — (A) Recently-activated CD8+ T cells are preferentially found at sites of parasite persistence such as heart and fat. Representative flow plots show surface expression of CD69 for cells isolated from indicated tissue during chronic (~230 dpi) T. cruzi infection. Plots gated on CD8+ T cells. Numbers in upper right indicate the percentage of CD69+ TSKB20+ cells; numbers in lower right indicate the percentage of TSKB20- cells expressing CD69. Data are representative of at least 2 independent experiments with n = 4–6 and depict mean+ SEM. * indicates percentage levels that are significantly different (* P ≤ 0.05, ** P ≤ 0.01, ***P ≤ 0.001) between specified groups. (TIF) [file ppat.1007410.s001.tif]

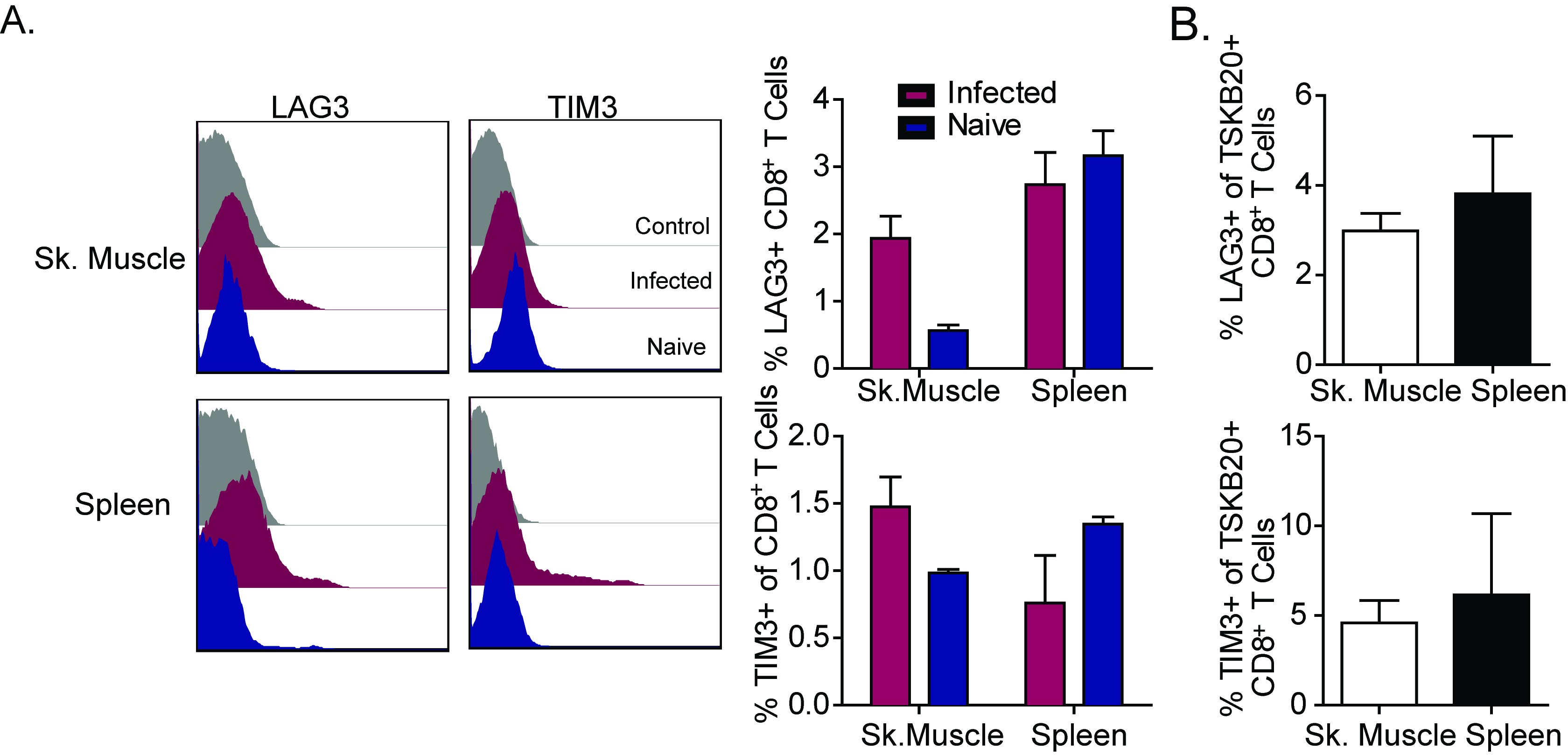

Supplement: S2 Fig — (A) Histograms display LAG-3 and TIM-3 expression by muscle and spleen CD44+ CD8+ T cells at >500 dpi from infected (magenta), isotype control (gray), naïve (blue) mice. The percentage of inhibitory receptor positive cells observed is described graphically for the total CD8+ and (B) TSKB20+ populations. (TIF) [file ppat.1007410.s002.tif]

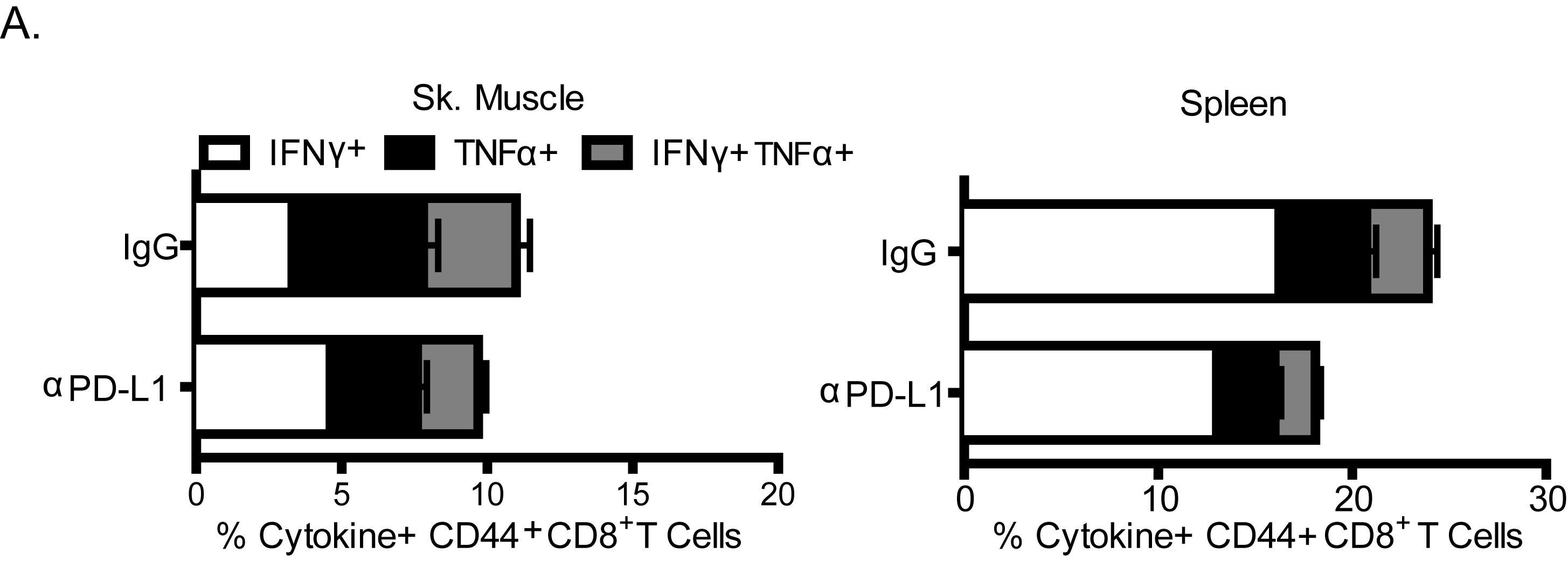

Supplement: S3 Fig — CD8+ T cells from chronically infected mice treated for 30 days with PD-L1 blocking antibody were stimulated for 5 hours with anti-mouse CD3ε. (A) The frequency of IFNγ+ (white), TNFα+ (black), and IFNγ+ and TNFα+ CD8+ T cells in the muscle (left) and spleen (right) is not increased by PD-L1 blockade. (TIF) [file ppat.1007410.s003.tif]

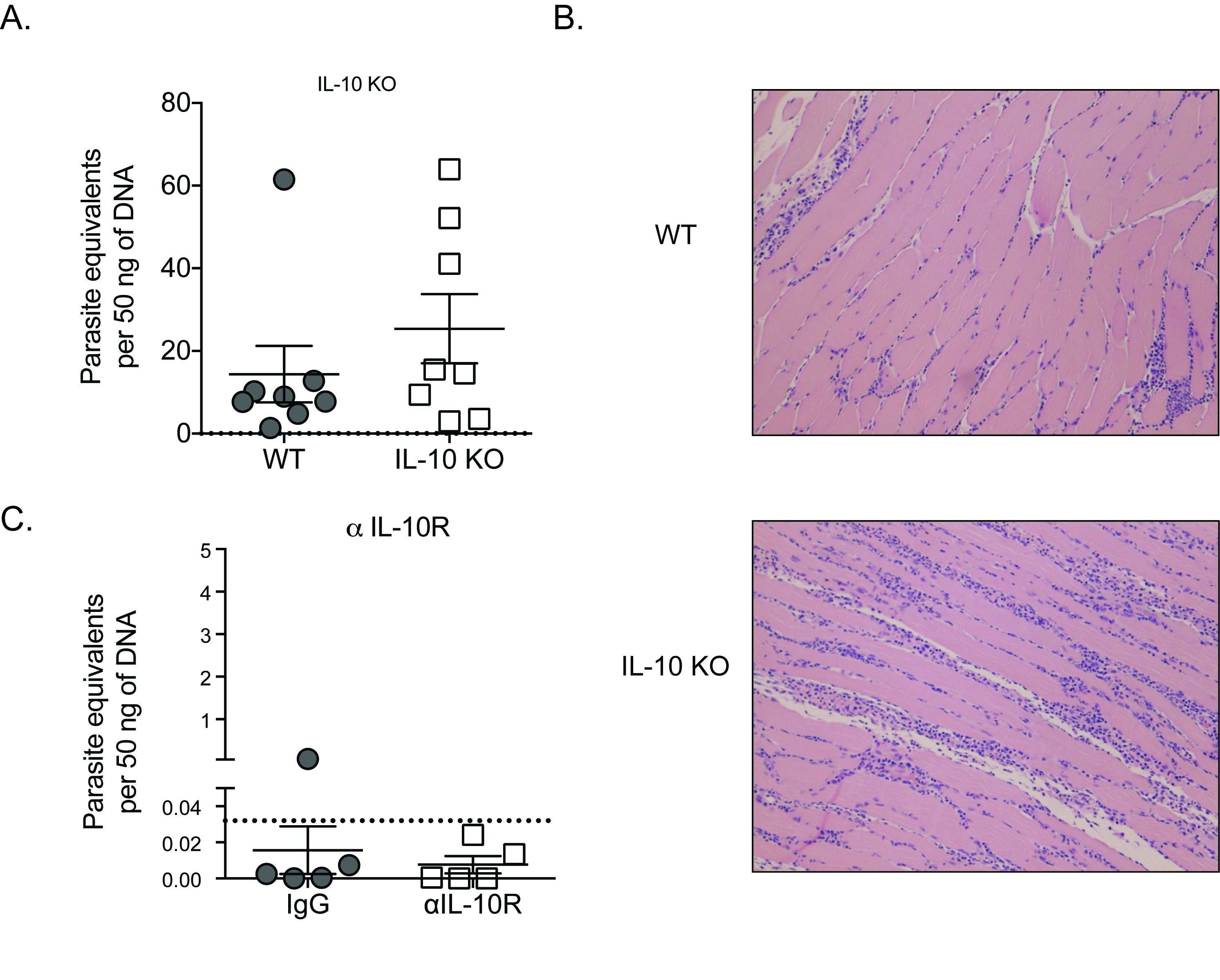

Supplement: S4 Fig — (A) IL-10 KO and WT mice exhibit similar parasite burden. Parasite load in skeletal muscle of IL-10 KO and WT mice during acute (30 dpi) T. cruzi infection was assessed by real-time PCR. (B) IL-10 KO mice cannot control the inflammatory response to T. cruzi. H&E sections of skeletal muscle from acutely infected IL-KO and WT mice. (C) Interrupting IL-10 signaling does not promote improved clearance of T. cruzi. Parasite loads in skeletal muscle of chronically T. cruzi-infected mice receiving anti-IL-10R Ab or rat IgG are plotted. The dashed line represents the limit of detection for quantitative real-time PCR. Bars show mean, which were not statistically different by Mann-Whitney test. Similar results were obtained in a repeat of each experiment. (TIF) [file ppat.1007410.s004.tif]
